# Bioinformatics Analysis and Immunogenicity Assessment of the Novel Multi‐Stage DNA Vaccine W541 Against *Mycobacterium Tuberculosis*

**DOI:** 10.1002/iid3.70074

**Published:** 2024-11-26

**Authors:** Yourong Yang, Yong Xue, Xiaoou Wang, Lan Wang, Jie Wang, Junxian Zhang, Yinping Liu, Yan Liang, Xueqiong Wu

**Affiliations:** ^1^ Beijing Key Laboratory of New Techniques of Tuberculosis Diagnosis and Treatment, Institute of Tuberculosis Research, Senior Department of Tuberculosis The Eighth Medical Center of PLA General Hospital Beijing China

**Keywords:** bioinformatic analysis, DNA vaccine, immunogenicity, simulated immunization, tuberculosis

## Abstract

**Background:**

Vaccination is one of the effective measures to prevent latent tuberculosis infection (LTBI) from developing into active tuberculosis (TB). Applying bioinformatics methods to pre‐evaluate the biological characteristics and immunogenicity of vaccines can improve the efficiency of vaccine development.

**Objectives:**

To evaluate the immunogenicity of TB vaccine W541 and to explore the application of bioinformatics technology in TB vaccine research.

**Methods:**

This study concatenated the immunodominant sequences of Ag85A, Ag85B, *Rv3407*, and *Rv1733c* to construct the W541 DNA vaccine. Then, bioinformatics methods were used to analyze the physicochemical properties, antigenicity, allergenicity, toxicity, and population coverage of the vaccine, to identify its epitopes, and to perform molecular docking with MHC alleles and Toll‐like receptor 4 (TLR4) of the host. Finally, the immunogenicity of the vaccine was evaluated in animal experiments.

**Results:**

The W541 vaccine protein is a soluble cytoplasmic protein with a half‐life of 1.1 h in vivo and an instability index of 45.37. It has good antigenicity and wide population coverage without allergenicity and toxicity. It contains 138 HTL epitopes, 73 CTL epitopes, 8 linear and 14 discontinuous B cell epitopes, and has a strong affinity for TLR4. Immune simulations have shown that it can effectively stimulate innate and adaptive immune responses. Animal experiments confirmed that the W541 DNA vaccine could effectively activate Th1‐ and Th17‐type immune responses, producing high levels of IFN‐γ and IL‐17A, but could not significantly increase antibody levels.

**Conclusion:**

The W541 DNA vaccine can induce strong cellular immune responses. However, further optimization of the vaccine design is needed to make the expressed protein more stable in vivo. Bioinformatics analysis could reveal the physicochemical and immunological information of vaccines, which is critical for guiding vaccine design and development.

## Graphical abstract

In this study, we construct the novel multi‐stage DNA vaccine, W541, against Mycobacterium tuberculosis. Then, bioinformatics methods were applied to analyze various physicochemical properties and immunological characteristics of the vaccine. Finally, the immunogenicity of the vaccine was evaluated in animal experiments. The results suggested that the W541 DNA vaccine can induce strong cellular immune responses, and further optimization of this vaccine is possible. Bioinformatics analysis could reveal vaccines’ physicochemical and immunological information, which is critical for guiding vaccine design and development.

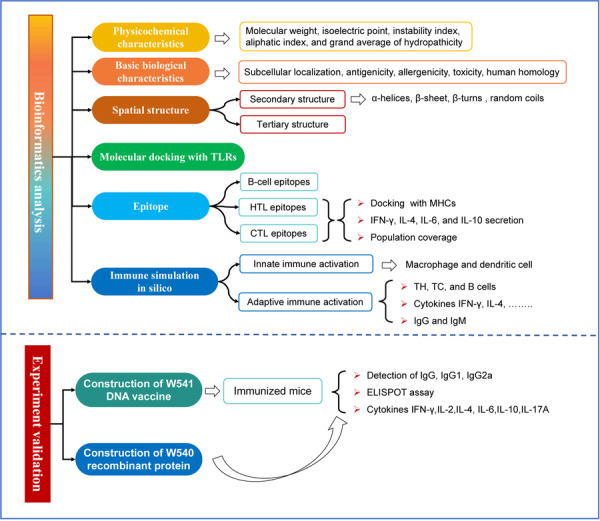

## 1 Introduction

Latent tuberculosis infection (LTBI) is characterized by the presence of specific immune responses to *Mycobacterium tuberculosis* (*M. tuberculosis*) in previously infected individuals without clinical evidence of active tuberculosis (TB) [1]. Currently, approximately 23% of the world's population is in an LTBI state, in which 5%–15% of those with LTBI may develop into active TB in their lifetime; LTBI has become a major source of active TB [2]. According to the 2015 WHO Guidelines for the Management of Latent Tuberculosis Infection, people with LTBI can take anti‐TB drugs to prevent them from developing active TB [2]. Considering that LTBI has no clinical symptoms, vaccination‐based prophylaxis seems more acceptable than chemotherapy. However, the Bacillus Calmette‐Guérin (BCG) vaccine, widely used for tuberculosis prevention, has a poor preventive effect on LTBI [3]. M72/AS01E, which was developed by GlaxoSmithKline Plc. and in phase IIb clinical trial, has a protective efficacy of only 54.0% against LTBI developing into active pulmonary TB [4]. The phase III clinical trial of the *M. vaccae* vaccine produced by Anhui ZhiFeiLongKeMa Biopharmaceutical Co., Ltd. showed a protective efficacy of 54.7% against LTBI [5]. These data suggest that developing an effective preventive and therapeutic vaccine against LTBI has broad prospects.

Nucleic acid vaccines represent the third revolution in the history of vaccine development. Compared with other types of vaccines, DNA vaccines offer the following advantages [1]: They can induce a comprehensive immune response, activating both humoral and cellular immunity, particularly inducing the production of cytotoxic T lymphocyte (CTL) responses that recognize *M. tb*‐infected cells and clear pathogens within them. This constitutes an effective pathway for eliminating *M. tb* lurking in macrophages, compensating for the weak CTL responses elicited by BCG vaccine, recombinant protein vaccines, and inactivated vaccines [2]. Vaccination with DNA vaccines is relatively safe, posing no risk of disease caused by bacterial virulence or residual virulent virus particles. It is safer for immunocompromised individuals than BCG [3]. Easy preparation and low production costs [4]. Plasmid DNA vaccines exhibit better stability and are easy to store and transport. Reports on the use of DNA vaccines to prevent or treat human diseases, such as AIDS [6], hepatitis B [7], hepatitis C [8], COVID‐19 [9], and tumor (including breast cancer, prostate cancer, and lymphoplasmacytic lymphoma) [10, 11, 12], among others, are gradually increasing. DNA vaccines have emerged as a hot topic in vaccine research, but so far, there is no research report on TB DNA vaccines in clinical trials. Currently, approximately 82.6% of the reported TB DNA vaccines in animal studies utilize a single *M. tb* antigen, 13.9% employ two or more different antigens or combinations of antigens and encoding adjuvants, and only 3.5% use antigen epitopes to construct vaccines [12]. In this study, we selected two immunodominant antigens (Ag85A and Ag85B), a latent activation antigen (Rv3407), and a dormant antigen (Rv1733c) from *M. tb* to construct a multi‐stage, multi‐antigen, and multi‐epitope DNA vaccine, hoping that it can be used not only for TB treatment but also for preventing the development of LTBI.

According to research reports, the Ag85 complex is the main secretory protein of *M. tb*, consisting of three proteins: Ag85A, Ag85B, and Ag85C. This complex accounts for approximately 30% of the total secreted protein in the *M. tb H37Rv* strain, and is readily isolated from early‐stage cultures. It has mycobacterial acid transferase activity, allowing trehalose to transfer and deposit on the cell wall of *M. tb*, playing an essential role in the final stage of *M. tb* cell wall synthesis [13, 14]. Ag85A and Ag85B contain multiple human T‐cell epitopes, and CD4^+^ T cells from TB patients could respond to the whole Ag85A or Ag85B polypeptides to produce interferon‐gamma (IFN‐γ) [15]. Our previous animal experimental studies [16, 17, 18, 19, 20]and the clinical trials reported [15, 20, 21, 22, 23, 24, 25, 26, 27, 28, 29, 30, 31, 32] have shown that Ag85A and Ag85B had high immunogenicity, could induce Th1‐type responses and cytotoxic T lymphocytes, reduce bacterial loads in lung and other tissues, alleviate lung lesions, and had better protective or therapeutic effects on TB or mouse model with latent tuberculosis infection (LTBI). At present, many new TB vaccines internationally choose Ag85A and/or Ag85B protein as vaccine antigens [22, 23], among which multiple vaccines have entered clinical trials, such as AERAS‐402 (including Ag85A, Ag85B, and TB10.4) [24], MVA85A\Ad5Ag85A\ChAdOx1 85A (all including Ag85A) [15, 20, 21, 22, 23, 24, 25, 26, 27], TB/FLU‐04L (including Ag85A and ESAT6) [28], GamTBvac (including Ag85A, ESAT6, and CFP10) [29], H1/IC31 (including Ag85B and ESAT6) [30], H4: IC31 (including Ag85B and TB10.4), H56: IC31 (including Ag85B, ESAT6, and *Rv2660c*) [31], AEC/BC02 (including Ag85B, ESAT6, and CFP10) [32].

*Rv3407* is a 99‐amino acid protein specifically expressed during the transition from dormancy to reactivation in *M. tb*. This protein is hypothesized to function as an antitoxin, exhibited low expression levels in virulent *M. tb* strains and was not detected in BCG strains. It may be a kind of antitoxin, only slightly expressed in *M. tb* virulent strains and not expressed in BCG strains [33, 34, 35]. Schuck D et al. found that the Rv3407 protein significantly induced abundant IFN‐γ and robust Th1‐type cellular immune responses in LTBI patients, whereas such responses were markedly absent in patients with active tuberculosis, indicating that the *Rv3407* protein may provide significant protection against dormant *M. tb* infection in susceptible populations [36]. Reece et al. engineered the *Rv3407* gene into the BCG vaccine and immunized mice with the recombinant BCG vaccine, and found that this modified BCG vaccine stimulated high levels of IFN‐γ production in mice and markedly enhanced protection against TB [37]. Our research group demonstrated through animal experiments that mice immunized with the *Rv3407* DNA vaccine could elevate levels of antigen‐specific IFN‐γ in the culture supernatants of splenic lymphocytes; this effect was accompanied by an increase in Th1 cell populations and an elevated Th1/Th2 cells ratio in the whole blood samples. Furthermore, the vaccine reduced the bacterial load in the lungs of mouse models with acute infection or LTBI and alleviated the degree of lung lesions [38, 39].

*Rv1733c* is a major dormancy antigen highly expressed by latent *M. tb* and can be well recognized by T cells from individuals with LTBI [40]. Zhang W et al. immunized mice with a DNA vaccine encoding *Rv1733c* and exhibited higher splenocyte stimulation index and IFN‐γ, IL‐2, and IL‐4 levels than those injected with saline [41]. Our research group used animal experiments to compare the preventive and therapeutic effects of MTB ag85ab and 7 types of LTBI DNA vaccines on a mouse LTBI model, which showed that the ag85ab, *rv2659c*, and *rv1733c* DNA vaccines reduced the bacterial load and degree of lung lesions in the mouse LTBI model [39]. Additionally, Coppola M et al. immunized mice with synthetic *Rv1733c* long peptides (28 amino acid sequences located at positions 57‐84, IPFAAAAGTAVQDSRSHVYAHQAQTRHP) and exhibited significantly increased expression of IFN‐γ, TNF‐α, and specific antibody, and reduced the pulmonary *M. tb* load. The findings suggest that *Rv1733c* has the potential for the prevention or treatment of TB [40].

On this basis, we selected the gene sequences encoding the full‐length of Ag85A antigen and 308 amino acids of Ag85B antigen as the vaccine backbone, added two epitope peptides of *Rv3407* protein (including 51 amino acids, specifically 15 amino acids at positions 16–30 and 36 amino acids spanning positions 61–96, respectively) and one epitope peptide of *Rv1733c* protein (28 amino acids at positions 57–84), and then concatenated them using the glycine‐rich flexible linker GSGGSG or GPGPG to construct a new TB DNA vaccine, named W541 based on the number of recombinant plasmids constructed by our research group over the years. The Ag85A and Ag85B proteins not only have strong immunogenicity but also act as carrier proteins for three epitope peptides, enhancing epitope peptide processing and immune presentation to elicit synergistic protective immunity against TB and LTBI.

The development of bioinformatics and the application of big data analytics have provided convenient conditions for the design and development of vaccines, allowing researchers to have the opportunity to understand vaccine‐related information in advance, thereby gaining a deeper understanding of vaccine characteristics and making corresponding optimizations to improve the efficiency of vaccine development [42, 43, 44, 45]. In this study, we used bioinformatics techniques to analyze various physicochemical properties and immunological characteristics of the W541 DNA vaccine. Then, we verified the immunogenicity of the W541 vaccine through animal experiments, exploring the feasibility of employing bioinformatics analysis methods as a means of preliminary assessment during tuberculosis vaccine development to aid vaccine research.

## 2 Materials and Methods

**Figure 1:**
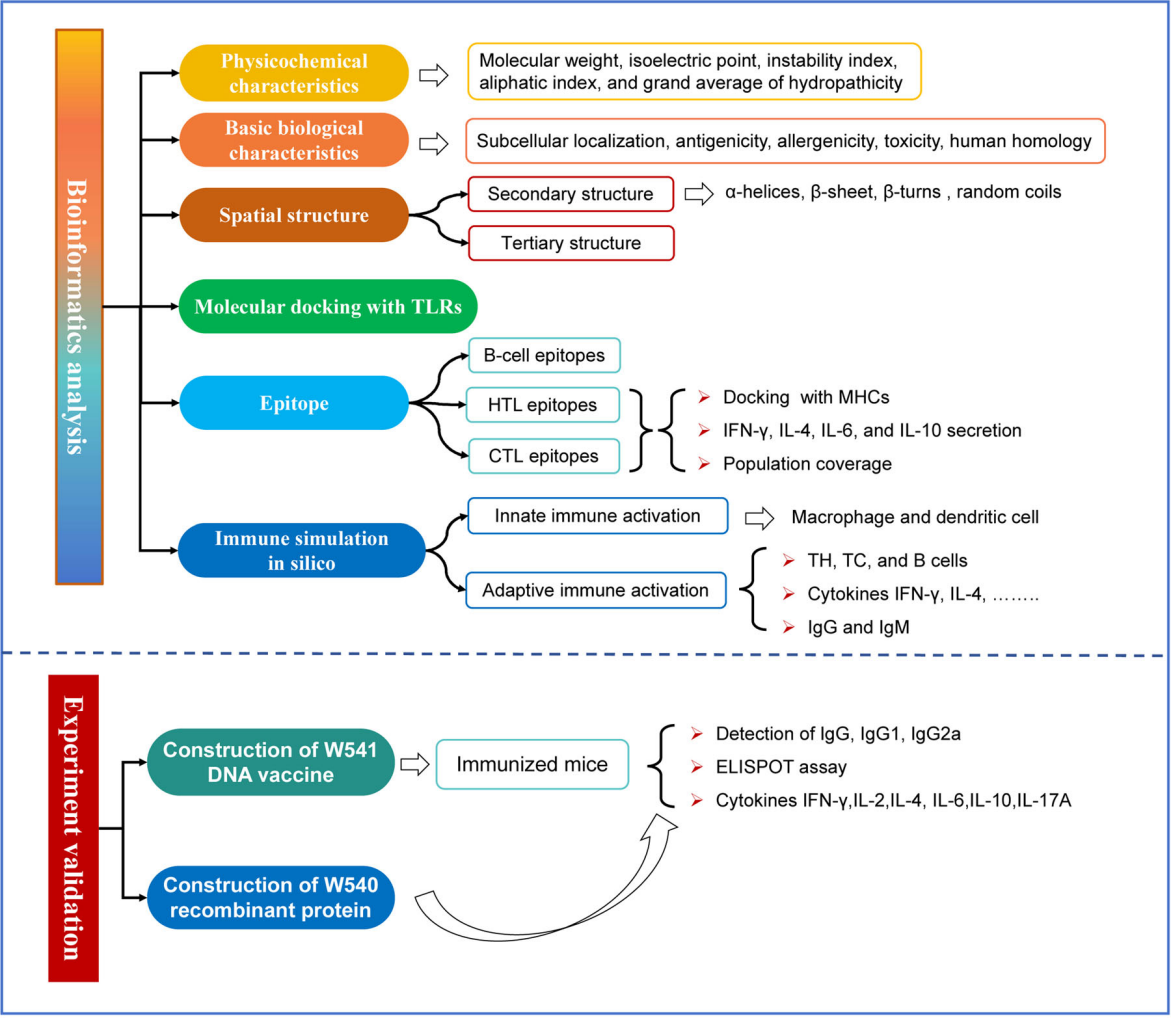
The flow chart of the study.

The flow chart of the study design is shown in Figure 1.

### 2.1 Bioinformatics Analysis

#### 2.1.1 The Amino Acid Sequence of the W541 Vaccine Protein

**Figure 2:**
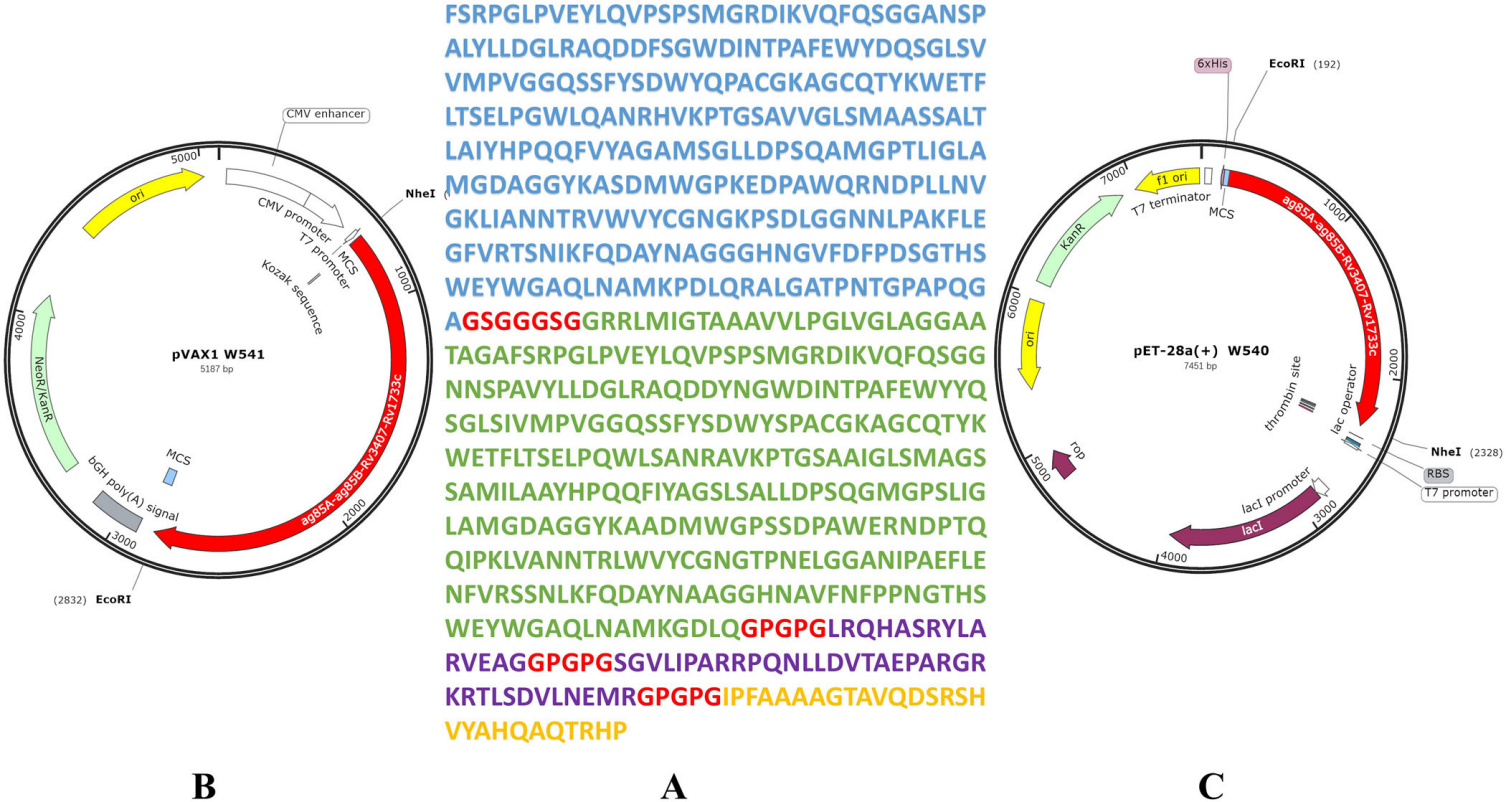
Construction of the W541 DNA vaccine and W540 recombinant protein plasmid. (A) The amino acid sequence of the W541 vaccine protein and the W540 recombinant protein is shown. In this sequence, blue characters represent amino acids from the Ag85A protein, green characters represent amino acids from the Ag85B protein, purple characters represent amino acids from the Rv3407 protein, yellow characters represent amino acids from the Rv1733c protein, and red characters indicate linker sequences. (B) Construction of the W541 recombinant DNA plasmid utilizing the pVAX1 vector. (C) Construction of the W540 recombinant protein plasmid using the pET28a vector.

The W541 vaccine protein contains immunodominant sequences of four antigens: ag85A, ag85B, *Rv3407*, and *Rv1733c*. The amino acid sequence of the vaccine protein is shown in Figure 2.

#### 2.1.2 The Physicochemical and Basic Biological Characteristics of the W541 Vaccine Protein

The physicochemical properties of the vaccine proteins, including amino acid composition, molecular weight, theoretical isoelectric point (pI), instability index (II), aliphatic index (AI), and grand average of hydropathicity (GRAVY), were predicted using the ProtParam server (https://web.expasy.org/protparam/) [46]. The SignalP 4.1 server (https://services.healthtech.dtu.dk/service.php?SignalP-4.1) [47], TMHMM 2.0 server (https://services.healthtech.dtu.dk/service.php?TMHMM-2.0), and the Hum‐mPLoc 2.0 module in the Cell‐PLoc 2.0 server (http://www.csbio.sjtu.edu.cn/bioinf/Cell-PLoc-2/) [48] were used to predict the presence of signal peptide sequences, transmembrane regions, and subcellular localization of the vaccine proteins after expression within host cells, respectively. The antigenicity, allergenicity, and toxicity of the vaccine proteins were predicted using the VaxiJen server (http://www.ddg-pharmfac.net/vaxijen/VaxiJen/VaxiJen.html) [49], AllerTop server (http://www.ddg-pharmfac.net/AllerTOP) [46], and ToxinPred server (https://webs.iiitd.edu.in/raghava/toxinpred/multi_submit.php) [50], respectively. Human homology analysis on the vaccine proteins was performed using the BLAST server(Organism:Humo sapiens) [51]. In the aforementioned prediction process, unless otherwise specified, the server's default parameters were used for prediction.

#### 2.1.3 The Spatial Structure Analyses of W541 Vaccine Protein and Molecular Docking With Toll‐like Receptor (TLR4)

Using the SOPMA server (https://npsa-prabi.ibcp.fr/cgi-bin/npsa_automat.pl?page=/NPSA/npsa_sopma.html) to predicte the secondary structure of the W541 vaccine protein [52]. Utilizing the bKunyun supercomputing platform to execute the AlphaFold2 [53] to predict the tertiary structure of W541, which was then authenticated using the Prosa server (https://prosa.services.came.sbg.ac.at/prosa.php) [54].

The GRAMM server (https://gramm.compbio.ku.edu/gramm) can systematically assess a series of docking postures between proteins and ligands and predict the most stable docking conformation [55]. First, the PDB files of TLR4 were downloaded from the PDB database (https://www.rcsb.org/) [56] and uploaded to the GRAMM server along with the W541 PDB files predicted by AlphaFold to predict the docking status between the TLR4 and W541 vaccine. Finally, the PDBePISA server (https://www.ebi.ac.uk/pdbe/pisa/) [57] was used to calculate the detailed docking data of the docking complexes, such as the interaction surface areas and binding energies. All of the above processes were analyzed using the servers' default parameters.

#### 2.1.4 Prediction of B‐Cell Epitopes in the W541 Vaccine Protein

The linear and discontinuous B‐cell epitopes of W541 vaccine proteins were predicted using the ElliPro server (http://tools.iedb.org/ellipro/) [58] with default parameters by uploading the AlphaFold‐predicted W541 PDB file.

#### 2.1.5 Prediction of HTL and CTL Epitopes in the W541 Vaccine Protein

The helper T lymphocytes (HTL) epitopes were predicted by the MHC‐II Binding Predictions server (http://tools.iedb.org/mhcii/) [59] using the “IEDB recommended method” specified for the Full HLA reference set with default parameters (completed in June 2023), which can predict epitopes for all 15 amino acid residues in the W541 vaccine protein. Then, the epitopes (excluding epitopes containing linker amino acids) with IC50 values below 500 nM were then further analyzed by the VaxiJen server with a threshold of 0.4, the IFN epitope server (http://crdd.osdd.net/raghava/ifnepitope/predict.php) [60], IL4pred server (https://webs.iiitd.edu.in/raghava/il4pred/predict.php) [61], IL6pred server (https://webs.iiitd.edu.in/raghava/il6pred/predict3.php) [62], and IL‐10pred server (https://webs.iiitd.edu.in/raghava/il10pred/predict3.php) [63] to predict their antigenicity, the ability to stimulate IFN‐γ, IL‐4, IL‐6, and IL‐10 secretion with default parameters.

The cytotoxic T lymphocytes (CTL) epitopes were predicted by the MHC‐I Binding Predictions server (http://tools.iedb.org/mhci/) [64] using the “recommended epitope predictor” specified for the default HLA allele reference set, which can predict all the 9 and 10 amino acid residues CTL epitopes of the vaccine. Additionally, the epitopes (excluding epitopes containing linker amino acids) with IC50 values below 500 nM were analyzed using the Class I Immunogenicity server (http://tools.iedb.org/immunogenicity/) with default parameters to predict their immunogenicity [65] and using the VaxiJen server to predict their antigenicity with default parameters.

Finally, all the HTL and CTL epitopes of the W541 vaccine protein were submitted to AllerTOP v. 2.0 server and ToxinPred server to predict their allergenicity and toxicity with default parameters.

#### 2.1.6 Analysis of Population Coverage and Molecular Docking of T‐Cell Epitopes With MHC **Molecule**s for the W541 Vaccine

All qualifying HTL epitopes and their corresponding MHC Restricted Alleles were submitted to the IEDB Population Coverage tool (http://tools.iedb.org/population/) [66], “World” and “Class II separate” options were selected to predict the global population coverage of HTL epitopes for the W541 vaccine. A similar method was used to predict the global population coverage of CTL epitopes for the W541 vaccine. The molecular docking between the vaccine epitopes and MHC molecules was performed using the GRAMM docking server. Firstly, the PDB files (5JLZ and 3OX8) of the MHC molecules were downloaded from the RCSB PDB database and processed with Pymol software 2.0 (an open‐source tool) to remove unnecessary ligands. Meanwhile, the structures of all docking epitopes were predicted using the PEP‐FOLD 3.5 server (https://mobyle.rpbs.univ-paris-diderot.fr/cgi-bin/portal.py#forms::PEP-FOLD3) [67]. The PDB files of MHC molecules and corresponding epitope structures were then submitted to the GRAMM docking server using the “free docking” mode. The docking results were analyzed using the PDBePISA server.

#### 2.1.7 Immune Simulation In Silico

The immune responses to the W541 vaccine were simulated using the C‐ImmSim server (http://150.146.2.1/C-IMMSIM/index.php) [68]. The simulation was configured with 540 steps, and the injection time steps were set to 1, 52, and 84, respectively, this configuration simulated vaccine administrations on Days 1, 14, and 28, modeling the subject's immune response from the initial injection through day 180.

All other parameters were maintained at their default settings.

### 2.2 Experiment Validation

#### 2.2.1 Preparation and Characterization of W541 DNA Vaccine and Its Corresponding Recombinant Protein W540

According to the genetic code rules, the amino acid sequence of the W541 vaccine protein (Figure 2A) was translated into a DNA sequence. The DNA sequences were then codon‐optimized to generate sequences specifically suited for eukaryotic and prokaryotic expression, and synthesized by Kwinbon Biotechnology Co., Ltd. (China). The synthesized eukaryotic expression DNA sequence and the pVAX1 vector plasmid were digested with restriction enzymes NheI and EcoRI at 37℃ for 2–4 h, respectively. The two fragments were ligated using DNA ligase overnight at 16℃ to construct the W541 plasmid (Figure 2B). The W541 plasmid was then transformed into *Escherichia coli* (*E. coli*) DH5α competent cells. Subsequently, the transformed *E. coli* DH5α cells were cultured in the LB medium at 37℃. After culturing, the W541 plasmid was isolated and purified using the plasmid extraction kit (Qiagen, Germany). Similarly, the synthesized prokaryotic expression DNA sequence and the pET28a vector plasmid were digested with restriction enzymes NheI and EcoRI at 37℃ for 2‐4 h, respectively. Then, the obtained fragments were ligated using DNA ligase overnight at 16℃ to construct the W540 recombinant protein plasmid (Figure 2C) and transformed into *E. coli* BL21 (DE3) competent cells. The transformed *E. coli* BL21(DE3) cells were cultured on LB medium and induced by 0.1 mM IPTG at 37℃ for 3 h before purifying the recombinant fusion protein [69]. The W541 plasmid was identified by sequencing and enzyme digestion. The W540 plasmid was identified by sequencing, and the protein expression of transformed *E. coli* BL21 (DE3) was identified by sodium dodecyl sulfate‐polyacrylamide gel electrophoresis (SDS‐PAGE) [70].

#### 2.2.2 Experimental Animals

Female BALB/c mice aged 56–62 days and weighing 18–20 g were obtained from Vital River Laboratory Animal Technology Co. Ltd. (Beijing, China). The animal experiments were conducted following the Regulations on Management of Experimental Animals formulated by the Ministry of Science and Technology of China. The Animal Ethics Committee of the Eighth Medical Center of PLA General Hospital has approved the experimental plan.

#### 2.2.3 Immunization of the Animals

Mice were divided into three groups (eight mice per group) and treated as follows: (1) Control group: each mouse was injected intramuscularly with 100 μL of saline; (2) pVAX1 group: each mouse was injected intramuscularly with 100 μg pVAX1 plasmid diluted in 100 μL saline; (3) Vaccine group: each mouse was injected intramuscularly with 100 μg W541 DNA vaccine diluted in 100 μL saline [39]. Mice received injections every 2 weeks for a total of three injections. At 5 weeks after the last immunization, the mice were euthanized. Blood samples were collected in heparin lithium anticoagulant tubes, and then the plasma was separated. The spleens of the mice were also harvested.

#### 2.2.4 Detection of specific antibodies against W540

The plasma was separated from the blood sample of each mouse and stored at −20℃. The enzyme‐linked immunosorbent assay (ELISA) was employed to individually detect specific antibodies IgG and its subtypes IgG1 and IgG2a against W540 protein in the plasma samples obtained from all 24 mice in three groups. The method is briefly described as follows: The ELISA plate (Costar, China) was coated with 100 μL 10 μg/mL W540 protein in the coating buffer (0.05 M NaHCO_3_, pH 9.6) and incubated overnight at 4℃. The plate was then washed with PBS‐T (1 × Phosphate‐Buffered Saline, pH7.4, containing 0.05% Tween‐20) and blocked with 200 μL/well of 2% (w/v) ovalbumin (Coolaber, China) in PBS buffer and incubated for 3 h at 37℃. Plasma samples were diluted 1:100 in assay buffer (1% w/v BSA in PBS‐T) and added in duplicate at 100 μL/well, followed by incubation for 2 h at 37℃. The coating buffer without W540 protein was added to each plate as a negative control. Goat anti‐mouse IgG, IgG1, and IgG2a conjugated to horseradish peroxidase (HRP) (Abcam, England) diluted 1:10,000 in assay buffer was added 100 μL/well and incubated for 1 h at 37℃. After washing, 100 μL of 3,3′,5,5′‐Tetramethylbenzidine substrate (BD, America) was added to each well, and plates were incubated in the dark at room temperature for 5 min. The enzymatic reaction was stopped by adding 50 μL 1 M H_2_SO_4_, and then the absorbance at 450 nm was measured using a microplate reader (Thermo, America).

#### 2.2.5 Detect the Number of Mouse Splenocytes Secreting IFN‐γ by Enzyme‐Linked Immunospot Assay (ELISPOT)

Equal amounts of mice spleen cells from the same group were mixed to reduce individual variations, and three independent experiments were subsequently conducted with the Mouse IFN‐γ ELISpotPLUS (HRP) assay kit (MABTECH AB, Sweden) to assess the immune response of mouse splenocytes to the W540 protein. The detailed process is as follows: 3 × 10^5^mixed cells were seeded in each well of the filter membrane plate pre‐packaged with IFN‐γ capturing antibody (MABTECH AB, Sweden). The negative control wells were added RPMI 1640 complete medium, while the positive control wells were added with phytohemagglutinin (PHA) (Sigma, USA) at a final concentration of 20 mg/mL. Following the kit's instructions, the number of splenocytes secreting IFN‐γ was measured after stimulation with W540 protein at a concentration of 30 µg/mL in a CO_2_ incubator at 37℃ for 20 h. The number of spots in each well was detected using CTL ImmunoSpot S5 Micro equipment (Cellular Technology, America).

### 2.3 Cytokine Analysis

Spleen cell suspensions from each group of mice were mixed in equal proportions to reduce individual variations, and then three repeated experiments were conducted. The mixed spleen cell concentration was adjusted to 3 × 10^6^ cells/mL. After stimulation with W540 protein at a final concentration of 30 µg/mL for 24 h, the supernatant from spleen cell cultures was subjected to analyze the expression levels of cytokine IFN‐γ, IL‐2, IL‐4, IL‐6, IL‐10, and IL‐17A using the CBA assay kit (BD Biosciences, USA) following the instructions provided. The measurements were performed in triplicate.

### 2.4 Statistical Analysis

The antibody detection, ELISPOT assay, and cytokine detection data were analyzed using GraphPad Prism 8 software. Normality analysis was performed using normality and lognormality tests. For data conforming to a normal distribution, the Brown‐Forsythe test and Tukey**'**s multiple comparison test were applied; for data not conforming to a normal distribution, the Kruskal‐Wallis test and Dunn's multiple comparison test were applied. The results were presented as mean ± standard error, and a *p*‐value of less than 0.05 was considered a statistically significant difference.

## 3 Results

### 3.1 Bioinformatics Analysis Results

#### 3.1.1 Physicochemical Characteristics and Basic Biological Characteristics of W541 Vaccine Protein

The W541 vaccine protein sequence contains 704 amino acid residues with a calculated molecular weight of 74.6 kDa and a theoretical isoelectric point of 6.29. In silico analysis exhibits that this protein has an average hydrophobicity of −0.294, an aliphatic index of 68.85, an instability index of 45.37, and possesses 57 potential phosphorylation sites and 7 potential glycosylation sites. Bioinformatics predictions indicate that the expressed vaccine protein lacks signal peptide and transmembrane domains, and has a half‐life of 1.1 h in mammalian reticulocytes, 3 min in yeast, and 2 min in *E. coli*. Subcellular localization prediction indicates that this protein expressed in human cells mainly localizes in the lysosomes and cytoplasm. These in silico predictions suggest that the W541 vaccine protein is a relatively large, soluble protein prone to degradation. In addition, computational analysis and prediction show that the W541 vaccine protein displays good antigenicity, nonallergenicity, nontoxicity, and no homology with human proteins (Table 1).

**Table 1:** Physicochemical and biological properties of the protein encoded by W541 DNA vaccine.

Properties	Results predicted
Total number of amino acids	704
Molecular weight	74,644.63
Formula	C3329H5035N923O993S24
Theoretical pI	6.29
Total number of negatively charged residues (Asp+Glu)	52
Total number of positively charged residues (Arg+Lys)	48
Total number of atoms	10304
Number of phosphorylation sites	57
Number of glycosylation sites	7
Estimated half‐life	1.1 h (mammalian reticulocytes, in vitro). 3 min (yeast, in vivo). 2 min (*Escherichia coli*, in vivo).
Instability index (II)	45.37 (unstable)
Aliphatic index (AI)	68.85
Grand average of hydropathicity (GRAVY)	−0.294
Antigenicity	0.6527 (ANTIGEN)
Allergenicity	Nonallergenicity
Toxicity	Nontoxin
Signal peptide	No
TMHMM result	Outside
Subcellular localization (Cell‐PLoc, Gram‐positive bacterial)	Extracell
Subcellular localization (Cell‐PLoc, Hum‐mPLoc)	Cytoplasm, lysosome

#### 3.1.2 The Spatial Structures of the W541 Vaccine Protein and Docking With TLR4

**Figure 3:**
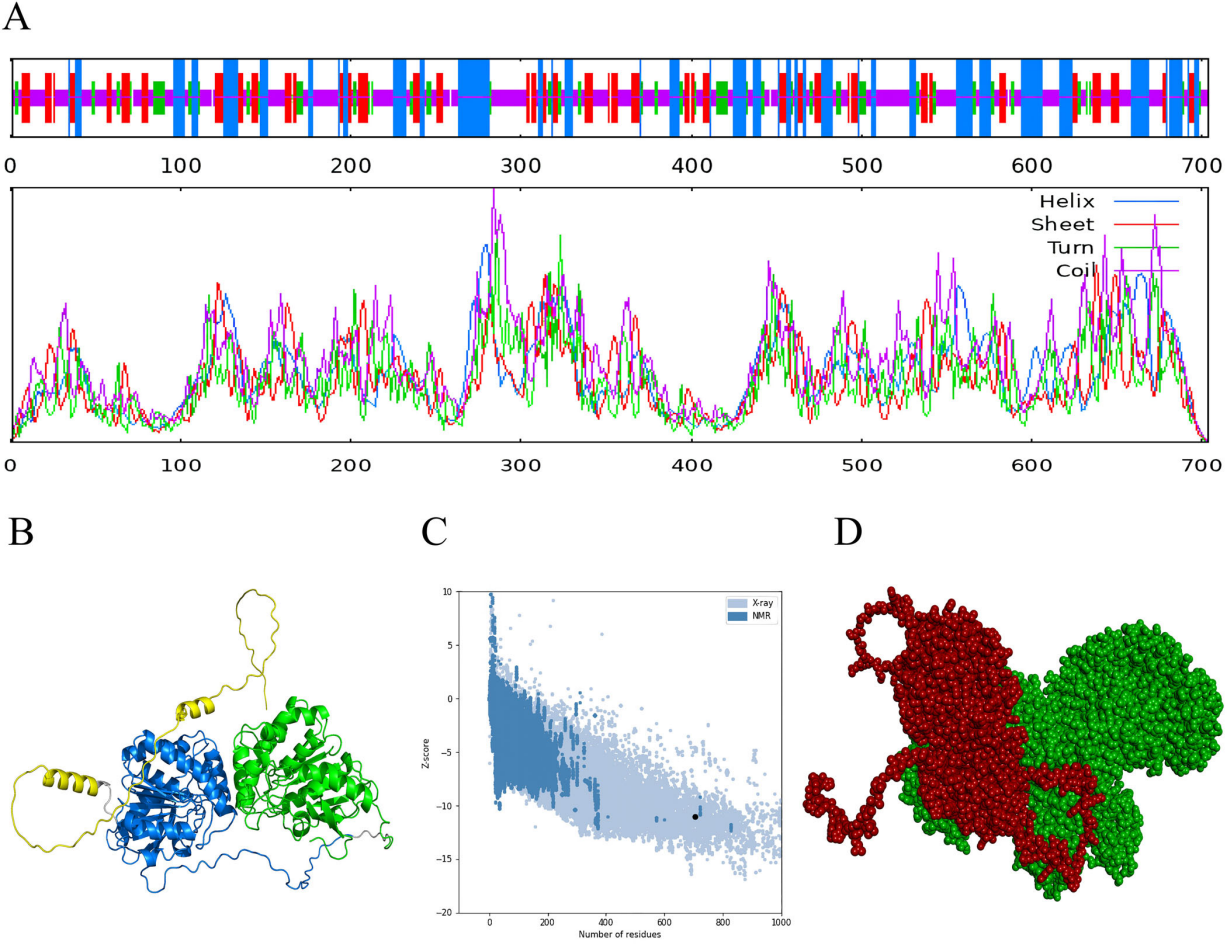
The spatial structures of the W541 vaccine protein and molecular docking with TLR4. (A) Prediction results of the secondary structure of the W541 vaccine protein. (B) The top‐ranked tertiary structure of the W541 protein predicted by AlphaFold2, where green represents the Ag85A domain, blue represents the Ag85B domain, and yellow represents the other three epitopes. (C) The score for the top‐ranked tertiary structure of the W541 vaccine protein provided by the Prosa server. The score falls within the blue or light blue range, suggesting high confidence and reliability of the predicted results. (D) The molecular docking results of W541 vaccine protein with TLR4, where the red part represents the vaccine protein and the green part represents TLR4.

According to the analysis conducted by the SOPMA server, the secondary structure of the W541 protein exhibited a distribution of α‐helices, β‐sheet, β‐turns, and random coils, accounting for 26.99%, 19.03%, 11.51%, and 42.47% of the total sequence, respectively. These four secondary structures were arranged alternately throughout the overall structure but had one distinct feature: the α‐helices primarily exist in the central and posterior parts of the protein (Figure 3A). Due to their inherently flexible nature, the random coils and β‐turns tend to be situated on the protein surface, showcasing a prominent structure. This region is usually enriched with epitopes that are advantageous for B‐cell recognition.

Tertiary structure prediction of the W541 vaccine protein using AlphaFold2 revealed five highly similar structural conformations. The top‐ranked tertiary structure type was visualized and is presented in Figure 3B. It showed that the Ag85A domain and Ag85B domain were folded separately, and three epitopes located at the C‐terminus exist independently as α‐helices and random coils. The Prosa server scored respectively the five predicted structures respectively as −10.99, −10.96, −10.65, −10.60, and −10.97, which fall within the expected range of the server's protein model database. This result indicates a high reliability of the predicted results (Figure 3C).

The top‐ranked tertiary structure type of W541 vaccine protein predicted by AlphaFold2 was selected to study the interaction with TLR4 to evaluate the immune system activation effect of the vaccine. The central region of the W541 vaccine protein showed a remarkable docking affinity with the B chain of TLR4, with a binding energy of −10.7 kcal/mol and an interface area of 2209.7 Å² (Figure 3D). The lower the binding energy indicates the closer vaccine‐TLR binding, the more likely it is to activate TLR4 and induce the production of cytokines and chemokines to enhance immune responses.

### 3.2 B‐Cell Epitopes of W541 Vaccine Protein

B‐cell epitopes can bind to B cell receptors (BCRs), activating downstream signaling cascades that regulate B‐cell activation and antibody production. Activated B cells can also act as antigen‐presenting cells. The prediction results from the ElliPro server showed that the W541 vaccine protein had 15 linear epitopes with prediction scores ranging from 0.511 to 0.755 and 11 discontinuous epitopes with prediction scores ranging from 0.543 to 0.987 (shown in Table S1).

### 3.3 HTL and CTL Epitopes of W541 Vaccine Protein

Based on the selection criteria, the W541 vaccine protein contained a total of 138 HTL epitopes (Table S2) and 73 CTL epitopes (Table S3). Of the 138 identified HTL epitopes, 101 showed the capacity to induce IFN‐γ production, 63 could induce IL‐4, 3 could induce IL‐6, and 19 could induce IL‐10 production. The W541 protein contained 10 HTL toxic epitopes and 6 CTL toxic epitopes. These toxic epitopes were concentrated within two discrete regions of the amino acid chain, totaling 21 residues (Table S4).

#### 3.3.1 Population Coverage Analysis and Molecular Docking of T Lymphocyte Epitopes With MHC for W541 Vaccine Protein

**Figure 4:**
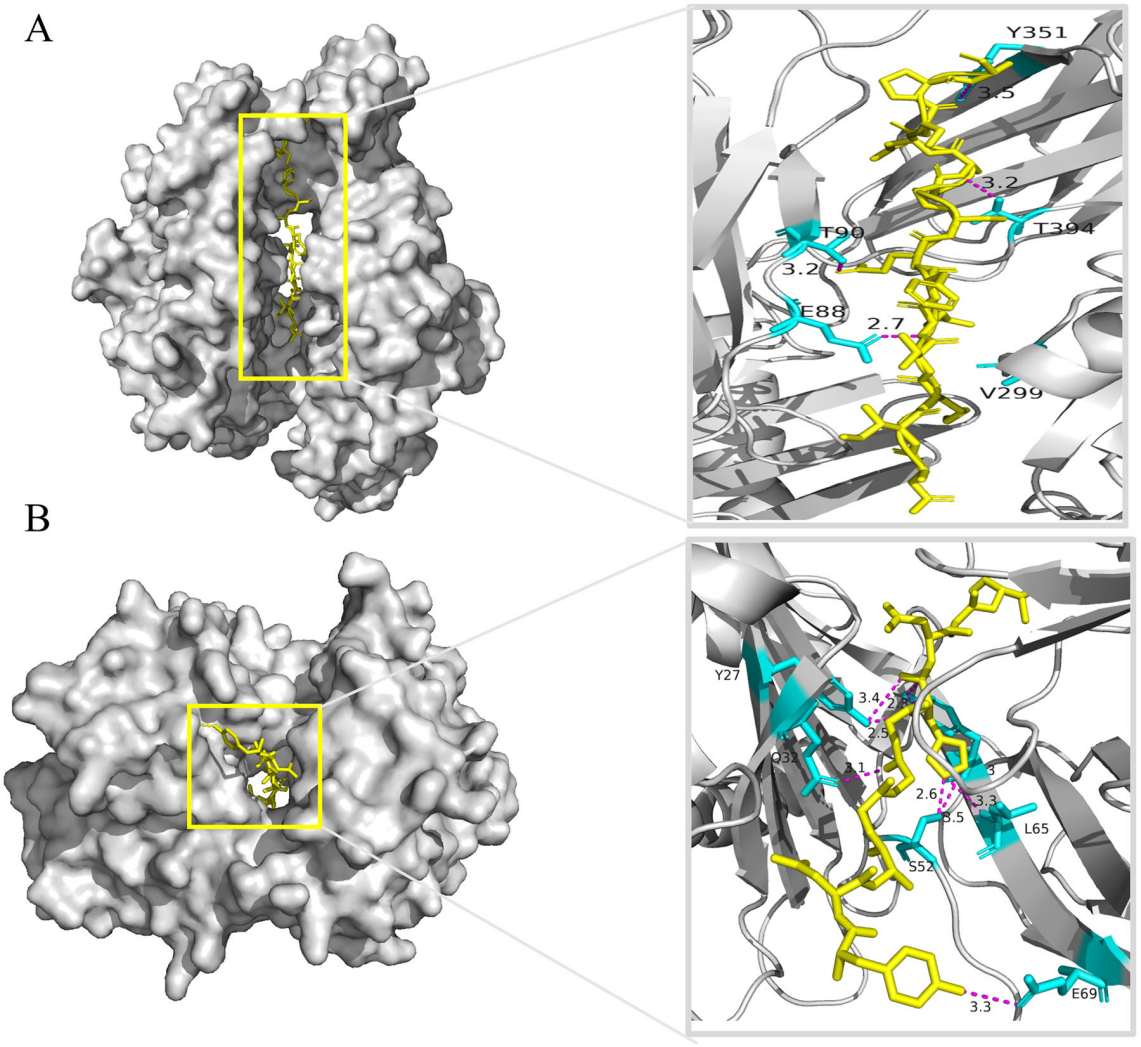
Molecular docking of two representative T‐cell epitopes with corresponding MHC molecules. (A) Molecular docking of HTL epitope DIKVQFQSGGANSPA with HLA‐DRB4*01:01 and (B) CTL epitope YNAGGGHNGV from Ag85A with HLA‐A*02:03. The yellow chains represent the epitopes, while the gray chains represent the MHC molecules. The blue structures represent the residues in the MHC molecule that form hydrogen bonds with the peptide chain, and the pink lines represent hydrogen bonds.

The population coverage analyses on the HTL and CTL epitopes of the W541 vaccine protein and their corresponding MHCs were performed using the Population Coverage server. The results revealed that the population coverage rates of HTL and CTL epitopes were 99.68% and 98.36%, respectively. 138 HTL epitopes that stimulate IFN‐γ secretion (Table S2) and 73 CTL epitopes (Table S3) could recognize 24 MHC class II molecules and 26 MHC class I molecules, respectively. Some of these epitopes had a binding affinity for a single MHC molecule, while others could bind to multiple MHC molecules. From this epitope library, 18 HTL epitopes (antigenicity threshold > 0.8, inducing only IFN, IC50 < 500) and corresponding MHC molecules are displayed in Table 2A; 27 CTL epitopes (antigenicity threshold > 0.8, class I immunogenicity > 0.1, IC50 < 500) and corresponding MHC molecules are shown in Table 2B. Molecular docking results of two selected T‐cell epitopes with their corresponding MHC molecules are shown in Figure 4.

**Table 2A:** Docking results of selected HTL epitopes with corresponding MHC alleles.

Antigens	Peptides	Alleles	Start	End	Antigenicity > 0.8	IFN induce	IL4 induce	IL6 induce	IL10 induce
Ag85A	EYLQVPSPSMGRDIK	HLA‐DRB1*04:01, HLA‐DRB1*04:01, HLA‐DRB1*01:01, HLA‐DRB1*01:01, HLA‐DRB1*04:05, HLA‐DRB1*04:05, HLA‐DRB1*07:01, HLA‐DRB1*07:01, HLA‐DRB1*09:01, HLA‐DRB1*09:01, HLA‐DRB5*01:01, HLA‐DRB5*01:01, HLA‐DRB3*01:01, HLA‐DRB3*01:01	9	23	0.9509	+	—	—	—
	YLQVPSPSMGRDIKV	HLA‐DRB1*04:01, HLA‐DRB1*04:01, HLA‐DRB1*01:01, HLA‐DRB1*01:01, HLA‐DRB1*07:01, HLA‐DRB1*07:01, HLA‐DRB5*01:01, HLA‐DRB5*01:01	10	24	1.5272	+	—	—	—
	QVPSPSMGRDIKVQF	HLA‐DRB1*03:01, HLA‐DRB1*03:01, HLA‐DRB4*01:01, HLA‐DRB4*01:01	12	26	1.595	+	—	—	—
	VPSPSMGRDIKVQFQ	HLA‐DRB1*03:01, HLA‐DRB1*03:01, HLA‐DRB4*01:01, HLA‐DRB4*01:01	13	27	1.6767	+	—	—	—
	PSPSMGRDIKVQFQS	HLA‐DRB1*03:01, HLA‐DRB1*03:01, HLA‐DRB4*01:01, HLA‐DRB4*01:01, HLA‐DQA1*01:02/DQB1*06:02, HLA‐DQA1*01:02/DQB1*06:02	14	28	1.483	+	—	—	—
	DIKVQFQSGGANSPA	HLA‐DRB1*04:01, HLA‐DQA1*05:01/DQB1*03:01, HLA‐DRB4*01:01, HLA‐DRB1*15:01, HLA‐DRB1*01:01	21	35	1.5849	+	—	—	—
	TGSAVVGLSMAASSA	HLA‐DQA1*01:02/DQB1*06:02, HLA‐DQA1*05:01/DQB1*03:01, HLA‐DRB1*01:01, HLA‐DRB1*08:02, HLA‐DRB1*04:01, HLA‐DRB1*09:01	118	132	0.9493	+	—	—	—
	VGLSMAASSALTLAI	HLA‐DRB1*09:01, HLA‐DQA1*05:01/DQB1*03:01, HLA‐DRB1*07:01, HLA‐DQA1*01:02/DQB1*06:02, HLA‐DRB3*02:02, HLA‐DPA1*01:03/DPB1*02:01, HLA‐DRB1*01:01, HLA‐DRB1*13:02, HLA‐DPA1*02:01/DPB1*01:01, HLA‐DRB1*03:01, HLA‐DRB1*04:01, HLA‐DRB1*04:05, HLA‐DRB1*15:01, HLA‐DRB5*01:01, HLA‐DRB4*01:01, HLA‐DPA1*03:01/DPB1*04:02	123	137	0.8465	+	—	—	—
	WVYCGNGKPSDLGGN	HLA‐DQA1*05:01/DQB1*03:01	208	222	1.4613	+	—	—	—
Ag85B	PGLVGLAGGAATAGA	HLA‐DQA1*05:01/DQB1*03:01, HLA‐DRB1*01:01, HLA‐DRB1*15:01, HLA‐DQA1*01:02/DQB1*06:02, HLA‐DRB1*09:01	317	331	0.8938	+	—	—	—
	LRAQDDYNGWDINTP	HLA‐DQA1*01:01/DQB1*05:01	373	387	1.2208	+	—	—	—
	KPTGSAAIGLSMAGS	HLA‐DQA1*05:01/DQB1*03:01, HLA‐DQA1*01:02/DQB1*06:02, HLA‐DRB1*01:01, HLA‐DRB1*09:01	447	461	0.9162	+	—	—	—
	PTGSAAIGLSMAGSS	HLA‐DQA1*05:01/DQB1*03:01, HLA‐DQA1*01:02/DQB1*06:02, HLA‐DRB1*09:01, HLA‐DRB1*01:01	448	462	1.0429	+	—	—	—
	TGSAAIGLSMAGSSA	HLA‐DQA1*05:01/DQB1*03:01, HLA‐DQA1*01:02/DQB1*06:02, HLA‐DRB1*01:01, HLA‐DRB1*09:01	449	463	1.2409	+	—	—	—
	GSAAIGLSMAGSSAM	HLA‐DQA1*05:01/DQB1*03:01, HLA‐DRB1*09:01, HLA‐DQA1*01:02/DQB1*06:02, HLA‐DRB1*01:01, HLA‐DRB1*07:01, HLA‐DRB1*04:01	450	464	0.9638	+	—	—	—
	SAAIGLSMAGSSAMI	HLA‐DQA1*05:01/DQB1*03:01, HLA‐DRB1*09:01, HLA‐DQA1*01:02/DQB1*06:02, HLA‐DRB1*01:01, HLA‐DRB1*07:01, HLA‐DRB1*04:01, HLA‐DRB5*01:01	451	465	0.863	+	—	—	—
	SMAGSSAMILAAYHP	HLA‐DQA1*01:02/DQB1*06:02, HLA‐DQA1*05:01/DQB1*03:01, HLA‐DRB1*15:01, HLA‐DRB1*12:01, HLA‐DRB1*07:01, HLA‐DRB1*01:01, HLA‐DRB1*09:01, HLA‐DRB5*01:01	457	471	0.8015	+	—	—	—
	DPSQGMGPSLIGLAM	HLA‐DQA1*05:01/DQB1*03:01, HLA‐DRB1*09:01, HLA‐DRB1*01:01	485	499	0.9049	+	—	—	—

**Table 2B:** Docking results of selected CTL epitopes with corresponding MHC alleles.

Antigens	Peptides	Alleles	Start	End	Length	Antigenicity > 0.8	Class I Immunogenicity > 0.1
Ag85A	FSGWDINTPA	HLA‐A*02:06, HLA‐A*02:01, HLA‐A*68:02, HLA‐A*02:03	48	57	10	0.9887	0.40528
	SGWDINTPA	HLA‐A*02:06	49	57	9	1.4382	0.24401
	SGWDINTPAF	HLA‐A*23:01, HLA‐B*15:01	49	58	10	1.5629	0.26756
	NTPAFEWYD	HLA‐A*68:02	54	62	9	0.927	0.4288
	ALTLAIYHP	HLA‐A*02:03	132	140	9	1.0436	0.1806
	FQDAYNAGGG	HLA‐A*02:06, HLA‐A*02:03, HLA‐A*68:02	240	249	10	1.4901	0.1219
	YNAGGGHNGV	HLA‐A*68:02, HLA‐A*02:06, HLA‐A*02:03	244	253	10	2.4651	0.15759
	NAGGGHNGV	HLA‐A*68:02	245	253	9	3.0386	0.12289
	WEYWGAQLN	HLA‐B*40:01, HLA‐B*40:01	264	272	9	0.904	0.18728
	RALGATPNTG	HLA‐A*02:06	280	289	10	0.8506	0.11318
Ag85B	GTAAAVVLP	HLA‐A*68:02	309	317	9	0.9815	0.15739
	VGLAGGAATA	HLA‐A*02:03	320	329	10	1.0136	0.1943
	GLAGGAATA	HLA‐A*02:03, HLA‐A*02:01, HLA‐A*02:06	321	329	9	1.3338	0.17233
	AGGAATAGAF	HLA‐B*15:01	323	332	10	1.1862	0.21456
	GGAATAGAF	HLA‐B*15:01	324	332	9	1.0961	0.17816
	GSAAIGLSM	HLA‐B*58:01, HLA‐B*15:01	450	458	9	1.2027	0.10755
	RLWVYCGNGT	HLA‐A*02:03	537	546	10	0.8136	0.10358
	TPNELGGAN	HLA‐B*35:01	546	554	9	1.3143	0.17121
	TPNELGGANI	HLA‐B*07:02	546	555	10	1.2042	0.18199
	ELGGANIPA	HLA‐A*68:02	549	557	9	0.8819	0.18295
	AYNAAGGHNA	HLA‐A*24:02	574	583	10	1.5728	0.16379
	YNAAGGHNAV	HLA‐A*68:02, HLA‐A*02:03, HLA‐A*02:06	575	584	10	1.6111	0.16592
	NAAGGHNAV	HLA‐A*68:02, HLA‐B*35:01, HLA‐A*02:06, HLA‐A*02:03	576	584	9	1.9957	0.12765
	NAAGGHNAVF	HLA‐B*15:01, HLA‐B*35:01	576	585	10	1.4758	0.16235
	AAGGHNAVF	HLA‐B*15:01, HLA‐B*35:01	577	585	9	1.1841	0.12765
	AVFNFPPNG	HLA‐A*30:01	583	591	9	0.9399	0.12191
Rv1733c	FAAAAGTAVQ	HLA‐B*15:01	679	688	10	0.8588	0.21685

### 3.4 Immune Simulation In Silico

#### 3.4.1 Activation of Innate Immune Cells by the W541 DNA Vaccine

**Figure 5:**
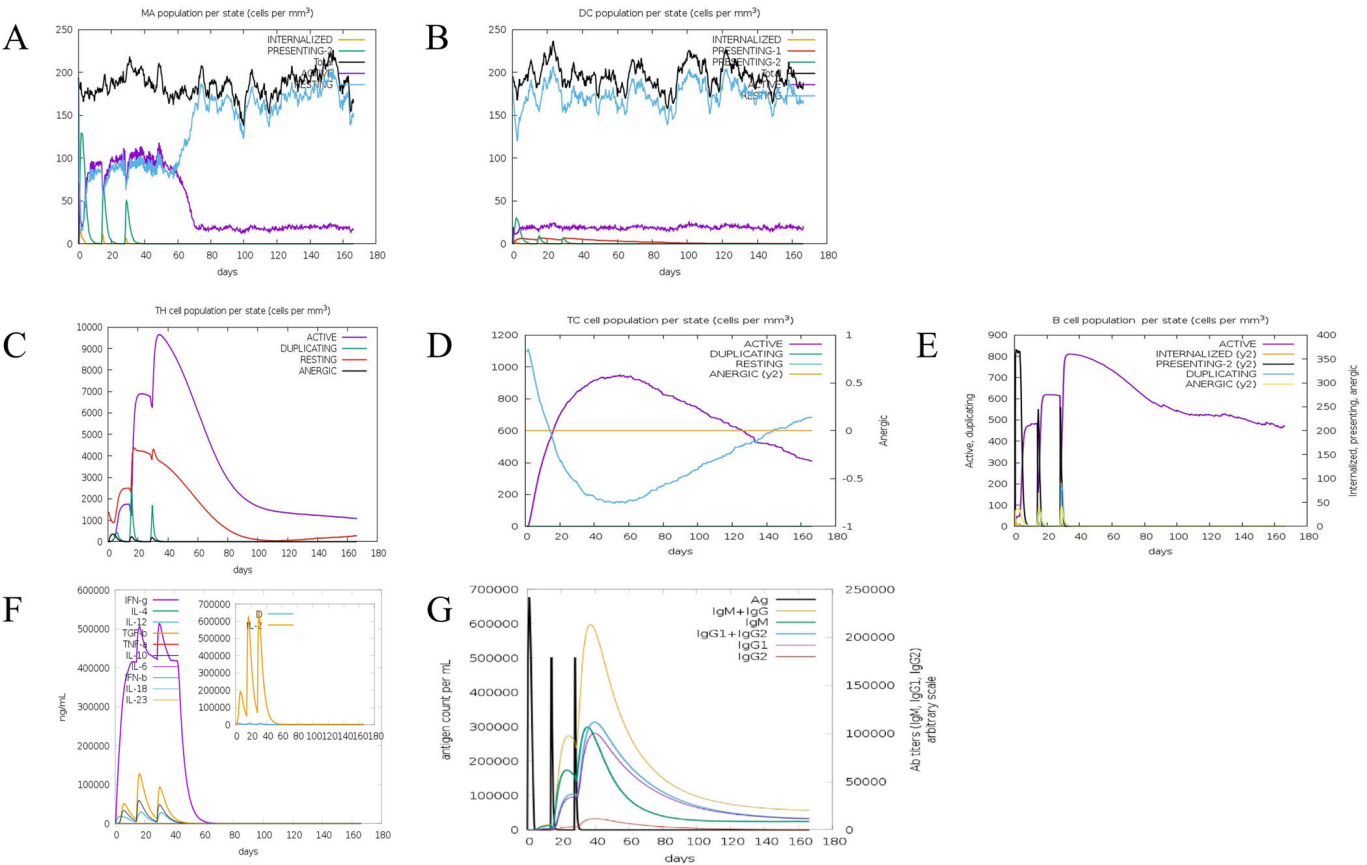
Activation of the native and adaptive immune system by the immune stimulation of the W541 DNA vaccine. (A) and (B) respectively represent the activation of macrophage (MA) and dendritic cell (DC) by W541; (C–E) respectively represent the activation of T helper cell (TH), cytotoxic T cell (TC), and B cells by W541; F and G respectively represent the secretion of cytokines and antibodies after W541 immunization.

Macrophages and dendritic cells are crucial to the body's innate immune responses. The C‐ImmSim server provides a simulation immune platform for evaluating the immune response after vaccination. After the immunization, the number of macrophages expressing MHC II (presenting‐2) significantly increased, but the magnitude of this increase gradually diminished with increasing immunization frequency. At the same time, the number of activated macrophages and resting macrophages significantly increased. the number of activated macrophages declined around day 50 after immunization and sharply decreased to a minimal level on Day 70 (Figure 5A). After each immunization, the number of presenting‐2 dendritic cells and dendritic cells expressing MHC I (presenting‐1) slightly increased, and the number of active dendritic cells only increased at a low level, while the number of resting dendritic cells increased significantly and remained at a high level (Figure 5B). The simulation results indicate a significant activation of macrophages and a relatively lower level of activation of dendritic cells after three immunizations of the W541 DNA vaccine.

#### 3.4.2 Activation of Adaptive Immune Cells by the W541 DNA Vaccine

The adaptive immune cells mainly include TH, TC, and B cells, which play a crucial role in anti‐TB immunity. After the initial vaccination, TH, TC, and B cells were activated. With each subsequent immunization, their activation was enhanced and reached their peaks on Day 35 for TH cells, Day 50 for TC cells, and Day 30 for B cells, respectively (Figure 5C–E). Following the first vaccination, IFN‐γ levels rapidly increased. With the second immunization, IFN‐γ levels were further elevated. After the third immunization, the IFN‐γ levels remained relatively stable compared to the second immunization and then rapidly declined, eventually returning to pre‐immunization levels around Day 50 (Figure 5F). A slight increase in IgG and IgM was observed after the initial immunization. After the second immunization, the expression of IgG and IgM increased rapidly and further increased after the third immunization. IgM peaked around Day 40, followed by a decline, and dropped to the plateau phase around Day 90. IgG peaked around day 50, followed by a similar decline, eventually reaching a steady phase around Day 150 (Figure 5G). Simulation results demonstrated that the W541 DNA vaccine could effectively activate TH, TC, and B cells, leading to elevated levels of corresponding cytokines such as IFN‐γ and antibodies IgG and IgM.

### 3.5 Experimental Validation Results

#### 3.5.1 Identification of W541 DNA Vaccine and W540 Recombinant Protein

**Figure 6:**
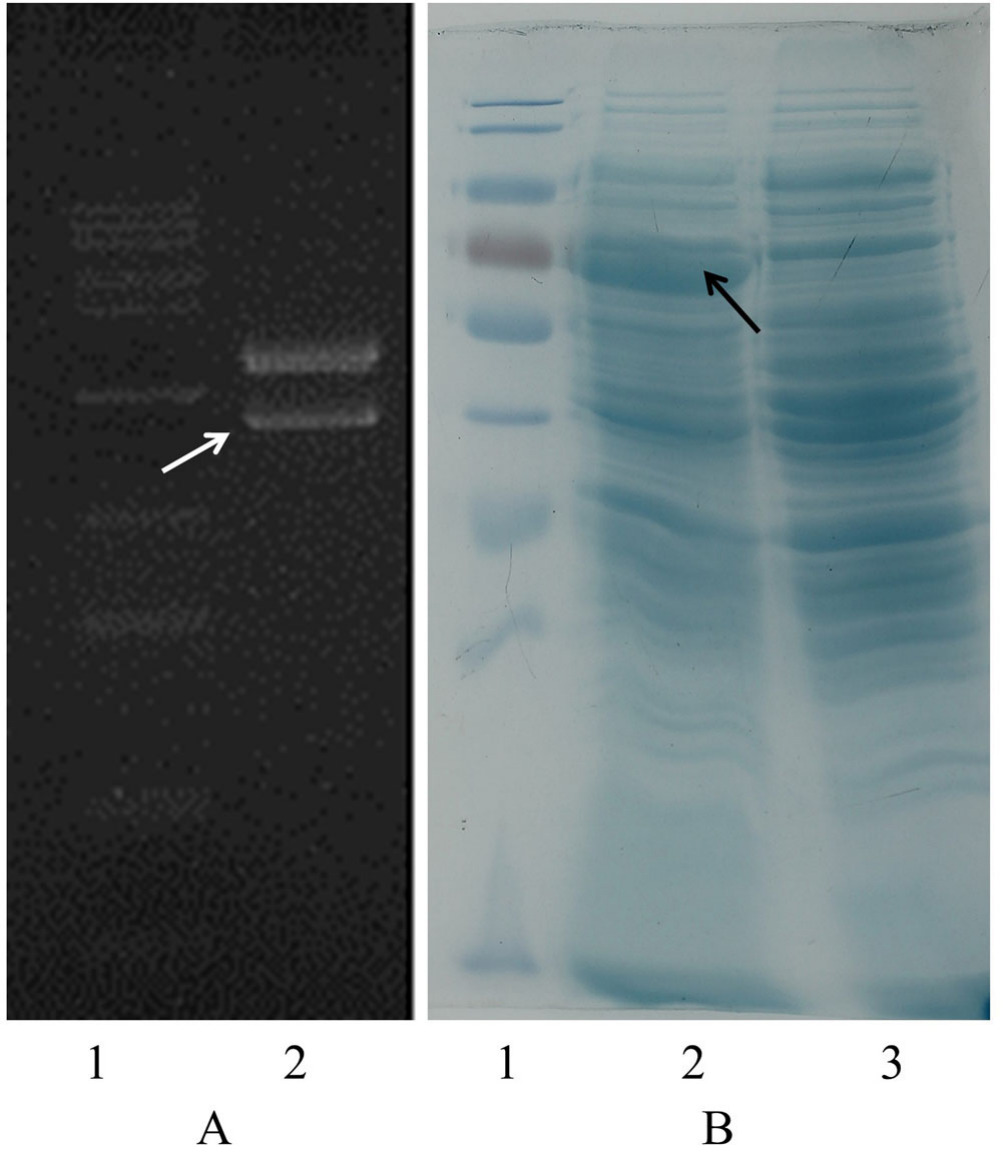
W541 plasmid DNA digested and expression of W540 recombinant protein A, 2% agarose gel electrophoresis. (A1) DNA molecular weight marker (15000, 12000, 10000, 7500, 4000, 2500, 1500, 1000, 500, 250 bp). (A2) The product of W541 recombinant plasmid DNA digested with restriction endonucleases NheI and EcoRI, a pVXA1 vector fragment band (2943 bp) and a W541 DNA fragment band (2244 bp) were observed. (B) 12% SDS‐PAGE analysis; (B1), a protein molecular weight marker (170, 130, 95, 72, 55, 43, 34, 26 kDa); (B2), the expression of W540 recombinant protein with an approximate molecular weight of 70 kDa in recombinant *E. coli* induced by IPTG at 37℃; B3, the expression of recombinant *E. coli* without IPTG induction at 37℃.

The sequencing findings indicate that the DNA sequences inserted in the W541 vaccine and W540 plasmid are congruent with the design. Upon induction with IPTG, the transformed *E. coli* BL21(DE3) with W540 plasmid expressed protein with an approximate molecular weight of 70 kDa on SDS‐PAGE (Figure 6).

#### 3.5.2 Antibody Production Induced by W541 DNA Vaccine

**Figure 7:**
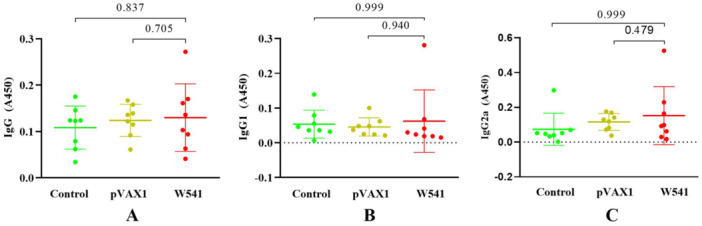
Antibody production in mice from the control, pVAX1, vaccine groups (A–C) show the levels of antibody IgG, IgG1, and IgG2a in the control group, pVAX1 group, and vaccine group (W541), respectively. All data are presented as the mean ± standard error. Normality testing revealed that the IgG data met the criterion (F‐statistic = 1.462, *p* > 0.05 for variance homogeneity), indicating equal variances among the groups, and thus Tukey's multiple comparison test was performed. In contrast, the IgG1 and IgG2a data did not follow a normal distribution, hence the Kruskal‐Wallis test and Dunn's test were applied. The results showed that the levels of IgG, IgG1, and IgG2a in the W541 vaccine group were not significantly different from those in the control group and the pVAX1 group (all *p* > 0.05).

After three immunizations, there was no significant difference in the antibody IgG, IgG1, and IgG2a levels among the W541 vaccine group, Control group, and pVAX1 group (*p* > 0.05) (Figure 7).

#### 3.5.3 Detect Number of Splenocytes Secreting IFN‐γ Using ELISPOT Assay

**Figure 8:**
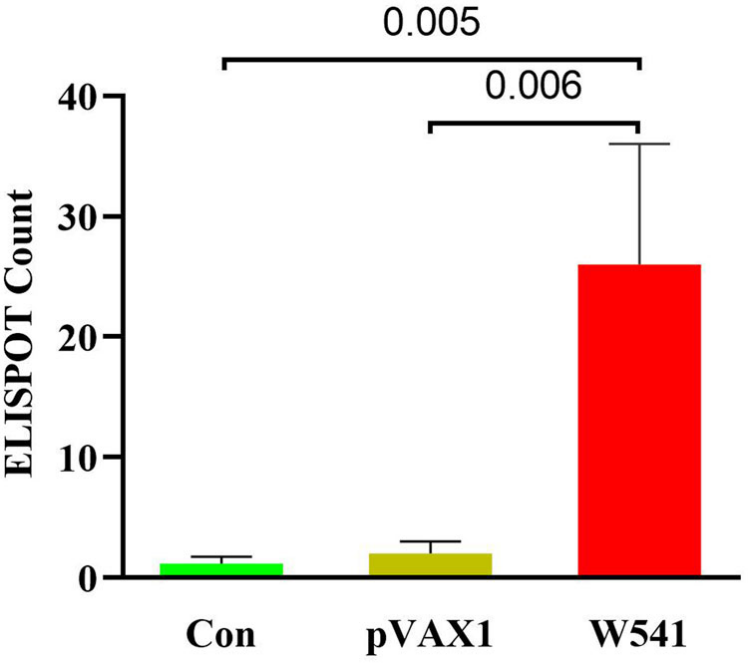
ELISPOT spot count of splenocytes secreting IFN‐γ: control, pVAX1, vaccine groups All data are presented as the mean ± standard error. The data met the normality criterion (*p* > 0.05). In the Brown‐Forsythe test, the F‐statistic was 3.363 (*p* > 0.05), indicating that the variances among the groups were equal. Compared with the control and pVAX1 groups, the W541 group showed significant differences (*p* < 0.05).

The ELISPOT assay results revealed that the number of spots produced by splenocytes secreting IFN‐γ in the vaccine group (26 ± 10) was significantly higher than that in the normal control group (1.2 ± 0.6, *p* < 0.01), as well as in the pVAX1 vector control group (2 ± 1, *p* < 0.01) (Figure 8). These compelling findings suggest that the W541 DNA vaccine effectively activated splenic lymphocytes in mice. Upon stimulation with the W540 protein, these activated splenocytes exhibited a rapid and robust secondary immune response.

#### 3.5.4 Analyses of Th1, Th2, and Th17 Cytokines in Splenocyte Culture Supernatants of Mice in Each Group

**Figure 9:**
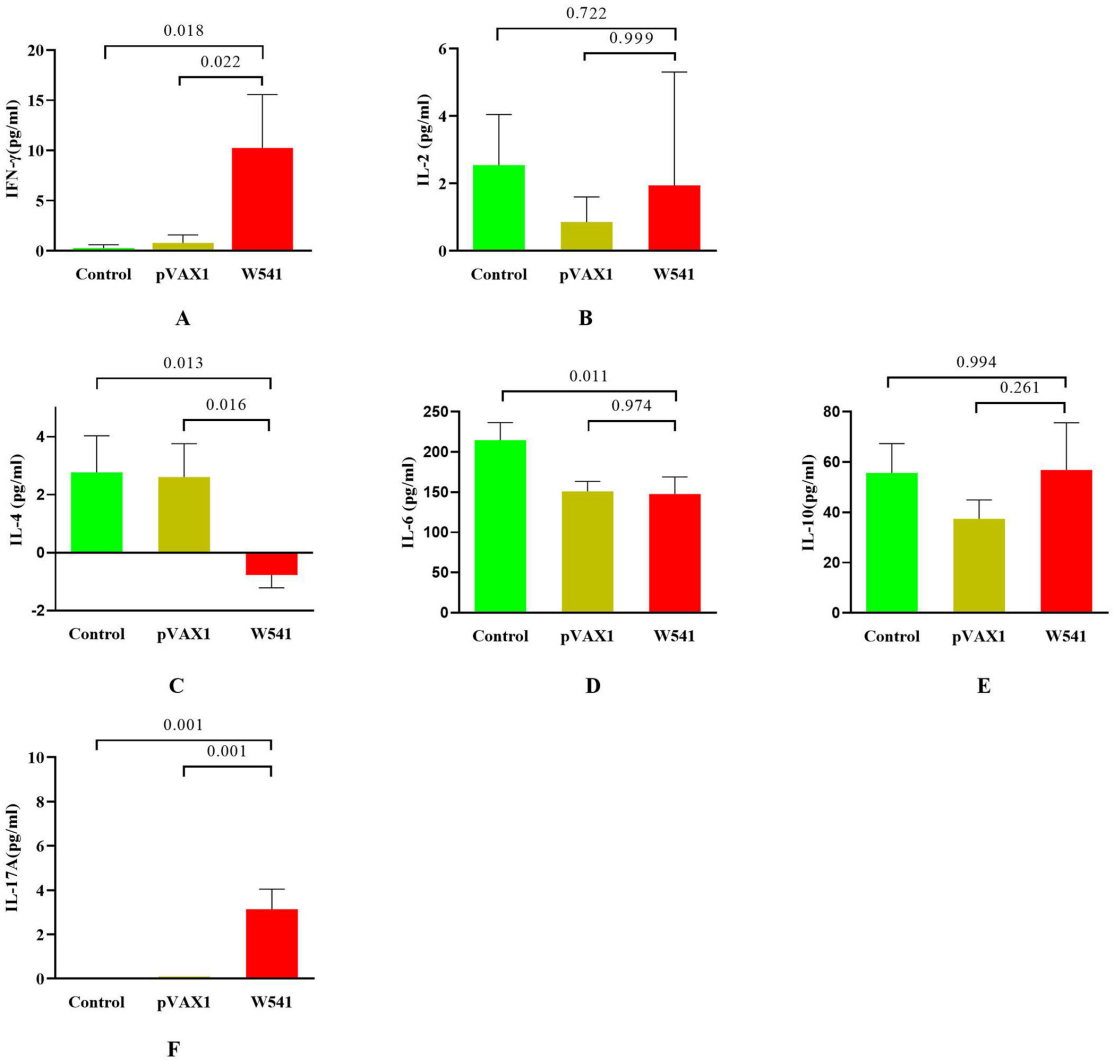
Levels of Th1, Th2, and Th17 cytokines in splenocyte culture supernatants (A) and (B) Th1‐type cytokines IFN‐γ and IL‐2; (C–E) Th2‐type cytokines IL‐4, IL‐6, and IL‐10; (F), Th17‐type cytokine IL‐17A. The IFN‐γ, IL‐4, IL‐6, IL‐10, and IL‐17A data met the normality criterion (*p* > 0.05) with an F‐statistic of 2.744, 0.9925, 0.1607, 0.2058, and 1.286 (*p* > 0.05), indicating equal variances among the groups, thus Tukey's multiple comparisons test was performed. The IL‐2 data did not follow a normal distribution, hence the Kruskal‐Wallis test and Dunn's test were performed.

Compared with the normal control group and the pVAX1 group, the levels of IFN‐γ and IL‐17 in the culture supernatants of splenocytes from mice in the W541 DNA vaccine group were significantly elevated (*p* < 0.05; Figure 9A, Figure 9F). However, there were no significant differences in IL‐2 levels among the vaccine and control groups (*p* > 0.05, Figure 9B). For Th2‐type cytokines, when comparing the W541 vaccine group with the normal control group, it was revealed that the W541 vaccine decreased expression of IL‐4 and IL‐6 by murine splenocytes (*p* < 0.05; Figure 9C, Figure 9D). There were no significant differences in IL‐10 expression among the three groups (*p* > 0.05, Figure 9E). These results demonstrate that the W541 DNA vaccine effectively activated a Th1‐type immune response in the host.

## 4 Discussion

In recent years, the rapid development of bioinformatics methods and the application of Internet databases have provided practical approaches for selecting protein‐dominant epitopes and the pre‐evaluation of vaccines [71, 72, 73], accelerating the vaccine research and development process. In this study, we employed bioinformatic methods to analyze the physicochemical properties, structure, safety, and immunological functions of a multi‐stage DNA vaccine W541 constructed by tandem immunodominant sequences of ag85A, ag85B, *Rv1733c*, and *Rv3407* antigens. Furthermore, we evaluated its immunogenicity through a murine model to verify the results of bioinformatics analysis and lay a foundation for further optimization of the vaccine.

The bioinformatic analysis revealed that the W541 vaccine protein was a soluble protein with a molecular weight of 74 kDa, exhibiting excellent antigenicity and broad population coverage, without allergenicity or toxicity. These fundamental findings strongly support the suitability of W541 as a vaccine candidate based on the essential criteria.

There is a close relationship between the secondary structure of a protein and B‐cell epitopes. The structures of irregular coiling and β‐turns, being more loosely arranged, are prone to distortion and spiralization, leading to their exposure to the protein surface. These regions typically harbor a greater abundance of B‐cell epitopes. The predictive analysis revealed that within the W541 vaccine protein, the sequence length of irregular coiling and β‐turns constitutes 53.98% of the total sequence length, most located on the surface of protein molecules. Furthermore, the protein contains 8 linear B‐cell epitopes and 14 discontinuous epitopes. These structures and epitopes provide the foundation for eliciting effective humoral immune responses in the host. The immune stimulation in silico revealed that the W541 vaccine protein could induce a heightened antibody response. However, in animal experiments, most mice in the W541 DNA vaccine group did not exhibit a significant increase in antibody levels. This phenomenon may be caused by the following reasons [1]: DNA vaccine type: Recent research has shown that mice immunized with DNA vaccines could easily induce strong cellular immune responses, but the antibody levels produced by DNA vaccines were usually weaker than those of BCG vaccine or corresponding protein vaccines administered with adjuvants [2, 74]. Method of immunization: Some tuberculosis DNA vaccines may not induce humoral immune responses when administered by intramuscular injection. The use of appropriate delivery systems, such as intramuscular electroporation, may improve the immunogenicity, protection or therapeutic efficacy of DNA vaccines [3, 74]. Subcellular localization and poor stability of expressed proteins: The subcellular localization of the vaccine‐expressed protein in the cytoplasm and lysosomes, and the poor stability of the W541 protein affects the vaccine's efficacy in eliciting a robust humoral immune response. The W541 vaccine protein expressed in the cytoplasm and lysosomes lost the opportunity to bind to BCR. In addition, it had a short half‐life and poor stability, indicating that the vaccine protein is prone to degradation, leading to loss of conformational epitopes, which is detrimental to B cell activation and antibody production [72]. Emerging evidence suggests that B cells and humoral immunity can regulate various immune responses of the intracellular pathogen *M. tuberculosis* [75]. In addition, unstable proteins may undergo degradation before being captured by macrophages and DC cells, compromising their antigen presentation effectiveness. Ultimately, this could also impact the activation of TH cells [73]. Therefore, optimizing the vaccine by increasing signal peptides to alter the subcellular localization of vaccine proteins or optimizing sequences to enhance their stability may help improve the immunogenicity of the vaccine [76, 77, 78]. Studies have shown that lysine residues exposed on protein surfaces can bind to ubiquitin and promote protein degradation by proteases [79, 80]. Additionally, the glycine, lysine, arginine, and cysteine residues at the N‐terminus and middle portion of proteins have marked effects on protein stability [81]. In the future, we will focus on studying DNA vaccine delivery methods (such as intramuscular electroporation and pulmonary delivery) and fine‐tuning the structure of the W541 vaccine by optimizing codons, using stability‐enhancing sequences, or exploring alternative delivery/formulation strategies to modify vaccine designs, to enhance protein stability and improve the immunogenicity of the vaccine protein.

TLRs play a crucial role in activating the anti‐TB immune responses, and TLR agonizts are considered a promising class of vaccine adjuvants. TLR4 is an essential receptor involved in MTB recognition within cells, expressed in both immune and nonimmune cells, and its structure includes the extracellular domain, transmembrane domain, and intracellular domain [82]. TLR4 forms homodimers to recognize pathogen‐associated molecular patterns such as lipopolysaccharides (LPS), lipoteichoic acid (LTA), dsRNA, etc. After TLR4 activation, it can initiate innate immune responses and regulate the migration, maturation, and function of antigen‐presenting cells while facilitating adaptive immune responses [83, 84]. *M. tb* dormancy‐related proteins *Rv2659c* and *Rv1738* can mediate the production of inflammatory cytokines through the TLR4 pathways [85]. Furthermore, the activated TLR4 can limit the survival of *M. tb* by inducing cellular autophagy [86]. These studies demonstrate the beneficial inclusion of TLR4 agonizts in the design of TB vaccines. The relatively stable binding between TLR and ligand is fundamental for TLR activation. Molecular docking simulations in this study have demonstrated that the expressed W541 protein in cells can establish stable interactions with TLR4, thereby possessing the potential to activate TLRs. Simulation immune results also proved that the W541 vaccine can effectively trigger the body's innate immune responses.

The prominent immune characteristics of TB patients include impaired Th1 cell‐mediated immune function or imbalanced Th1/Th2 cell immune responses, which represent the primary risk factors of TB [87, 88]. Hence, effectively activating the host's Th1‐type cell‐mediated immune response is essential for TB vaccines to exert their protective effects against *M. tb* [89]. IFN‐γ plays a crucial role in the defense against *M. tb* infection by promoting the proliferation and differentiation of Th0 cells into Th1 cells and activating macrophages [90]. The bioinformatics analysis of this study showed that the W541 vaccine protein was an antigen with a majority of T‐cell epitopes, containing a total of 138 HTL epitopes, in which 101 HTL epitopes could induce the production of IFN‐γ. In addition, it also included a smaller number of HTL epitopes that could induce the production of IL‐4, IL‐6, and IL‐10. The simulated immunization with the W541 vaccine demonstrated that the W541 DNA vaccine could effectively activate TH cells and elicit a robust release of IFN‐γ and small amounts of TGF‐β, IL‐10, and IL‐12 secretion. In contrast, the secretion of other cytokines (including IL‐4 and IL‐6) was not observed. Animal experiments have confirmed that mice in the vaccine group exhibited significantly higher levels of IFN‐γ secretion in spleen cells compared to the control group, and there was no significant increase in the secretion levels of Th2‐type cytokines (IL‐4, IL‐6, and IL‐10). These results collectively validate the consistency between most of the immunoinformatics analysis results and animal experimentation results of the vaccine, demonstrating a favorable structure‐function relationship. IL‐2 is also a representative multifunctional cytokine mainly secreted by CD4^+^ T cells in the Th1‐type immunity, which can activate T cells and promote cytokine production, activate macrophages, enhance the killing activity of NK cells, and promote the production of immunoglobulins by B cells, playing an essential role in the body's anti‐TB immunity. In this study, immunoinformatics prediction showed a significant increase in IL‐2, but animal experiments exhibited that the W541 immunization could not effectively induce the production of IL‐2. The possible reasons for this are [1]: W541 immunization expressed high levels of antigens in mice, mainly inducing effector memory T cells (TEM) and producing predominant high IFN‐ γ/low IL‐2 reaction. After weakened vaccine expression, it may mainly induce central memory T cells (TCM), possibly with a predominant low IFN‐ γ/high IL‐2 reaction [2]. It may also be because mouse spleen cells were stimulated by the specific antigen W540 for a shorter time (24 h), which generally requires culture for 72 h to induce high levels of IL‐2 production [91]. In addition, the experiment results showed that the level of IL‐17A in the vaccine group was significantly higher compared to the control group. IL‐17A was produced by activated T cells and mediated the production of inflammatory molecules, chemokines, antimicrobial peptides, and remodeling proteins [92], playing an essential role in the immune response to *M. tb* [93]. The W541 vaccine protein contains 138 CTL epitopes, which can effectively activate cytotoxic T cells, confirmed in immune simulations. Cytotoxic T cells are crucial in clearing *M. tb* infection by promoting target cell apoptosis or clearing infected target cells and persisting bacteria through the perforin‐granzyme pathway [94, 95, 96, 97]. Numerous HTL and CTL epitopes within the W541 vaccine protein have been experimentally validated. For instance, KLIANNTRV has been identified as an HLA‐A2‐specific CD8^+^ immunodominant antigen peptide [98], PBMCs from TB patients exhibit a strongly proliferative response to a peptide (DQSGLSVVMPVGGQSSFY) derived from Ag85 [99].

## 5 Conclusions

In summary, the results of the bioinformatics analysis showed a strong positive correlation with animal experiments, indicating that bioinformatics analysis can provide valuable prospective information for vaccine research. The study also showed that the W541 DNA vaccine, composed of the sequences encoding the Ag85A, Ag85B, Rv3407, and Rv1733c antigens, contains a large number of HTL and CTL epitopes that can activate TH cells and TC cells, and induce mainly Th1 and Th17 immune responses in the body. However, because the vaccine is administered by intramuscular injection and the protein expressed by the vaccine is not very stable in vivo, W541 could not effectively induce humoral immune responses. Therefore, we will improve vaccine immunization methods and optimize vaccine design to address the issues with W541, and further evaluate the immunogenicity and protective efficacy of the vaccine against LTBI in a mouse LTBI model.

## Author Contributions

**Yourong Yang:** data curation, investigation, methodology, resources, writing–original draft. **Yong Xue:** data curation, methodology, validation. **Xiaoou Wang:** validation. **Lan Wang:** validation. **Jie Wang:** validation. **Junxian Zhang:** validation. **Yinping Liu:** validation. **Yan Liang:** investigation, methodology, validation. **Xueqiong Wu:** funding acquisition, methodology, supervision, writing–review and editing.

## Ethics Statement

The animal study was reviewed and approved by The Animal Ethical Committee of the Eighth Medical Center of the Chinese PLA General Hospital (Approved Number: 309202108250973).

## Conflicts of Interest

The authors declare that the research was conducted without any commercial or financial relationships that could be construed as a potential conflict of interest.

## Supporting information

Supporting information.

Supporting information.

Supporting information.

Supporting information.

## Data Availability

The original contributions presented in the study are included in the article and Supplementary Material. Further inquiries can be directed to the corresponding author.

## References

[1] H. Getahun , A. Matteelli , I. Abubakar , et al., “Management of Latent Mycobacterium Tuberculosis Infection: Who Guidelines for Low Tuberculosis Burden Countries,” European Respiratory Journal 46, no. 6 (2015): 1563–1576. 26405286 10.1183/13993003.01245-2015 PMC4664608

[2] R. M. G. J. Houben and P. J. Dodd , “The Global Burden of Latent Tuberculosis Infection: A Re‐Estimation Using Mathematical Modelling,” PLoS Medicine 13, no. 10 (2016): e1002152. 27780211 10.1371/journal.pmed.1002152 PMC5079585

[3] A. H. Alvarez and M. A. Flores‐Valdez , “Can Immunization With Bacillus Calmette‐Guérin Be Improved for Prevention or Therapy and Elimination of Chronic Mycobacterium Tuberculosis Infection?,” Expert Review of Vaccines 18, no. 12 (2019): 1219–1227. 31826664 10.1080/14760584.2019.1704263

[4] O. Van Der Meeren , M. Hatherill , V. Nduba , et al., “Phase 2b Controlled Trial of M72/AS01(E) Vaccine to Prevent Tuberculosis,” New England Journal of Medicine 379, no. 17 (2018): 1621–1634. 30280651 10.1056/NEJMoa1803484 PMC6151253

[5] J. J. Mao , X. Zang , W. L. Yue , et al., “Population‐Level Health and Economic Impacts of Introducing Vaccae Vaccination in China: A Modelling Study,” BMJ Global Health 8, no. 5 (2023): e012306. 10.1136/bmjgh-2023-012306 PMC10254880 37257938

[6] G. J. Wilson , B. Rodriguez , S. S. Li , et al., “Cellular and Humoral Responses to an HIV DNA Prime by Electroporation Boosted With Recombinant Vesicular Stomatitis Virus Expressing HIV Subtype C ENV in a Randomized Controlled Clinical Trial,” Vaccine 41, no. 16 (2023): 2696–2706. 36935288 10.1016/j.vaccine.2023.03.015 PMC10102555

[7] F. Q. Yang , G. R. Rao , G. Q. Wang , et al., “Phase IIb Trial of In Vivo Electroporation Mediated Dual‐Plasmid Hepatitis B Virus DNA Vaccine in Chronic Hepatitis B Patients Under Lamivudine Therapy,” World Journal of Gastroenterology 23, no. 2 (2017): 306–317. 28127204 10.3748/wjg.v23.i2.306 PMC5236510

[8] J. M. Jacobson , D. Zahrieh , C. A. Strand , et al., “Phase I Trial of a Therapeutic DNA Vaccine for Preventing Hepatocellular Carcinoma from Chronic Hepatitis C Virus (HCV) Infection,” Cancer Prevention Research 16, no. 3 (2023): 163–173. 36534786 10.1158/1940-6207.CAPR-22-0217 PMC9992130

[9] L. Aurisicchio , N. Brambilla , M. E. Cazzaniga , et al., “A First‐In‐Human Trial on the Safety and Immunogenicity of Covid‐Evax, a Cellular Response‐Skewed Dna Vaccine against COVID‐19,” Molecular Therapy 31, no. 3 (2023): 788–800. 36575794 10.1016/j.ymthe.2022.12.017 PMC9792419

[10] M. L. Disis , K. A. Guthrie , Y. Liu , et al., “Safety and Outcomes of a Plasmid DNA Vaccine Encoding the ERBB2 Intracellular Domain in Patients With Advanced‐Stage ERBB2‐Positive Breast Cancer: A Phase 1 Nonrandomized Clinical Trial,” JAMA Oncology 9, no. 1 (2023): 71–78. 36326756 10.1001/jamaoncol.2022.5143 PMC9634596

[11] D. G. McNeel , H. Emamekhoo , J. C. Eickhoff , et al., “Phase 2 Trial of a DNA Vaccine (pTVG‐HP) and Nivolumab in Patients With Castration‐Sensitive Non‐Metastatic (M0) Prostate Cancer,” Journal for Immunotherapy of Cancer 11, no. 12 (2023): e008067. 38101860 10.1136/jitc-2023-008067 PMC10729272

[12] S. J. Szymura , L. Wang , T. Zhang , et al., “Personalized Neoantigen Vaccines as Early Intervention in Untreated Patients With Lymphoplasmacytic Lymphoma: A Non‐Randomized Phase 1 Trial,” Nature Communications 15, no. 1 (2024): 6874. 10.1038/s41467-024-50880-2 PMC11317512 39128904

[13] X. Tang , W. Deng , and J. Xie , “Novel Insights Into Mycobacterium Antigen Ag85 Biology and Implications in Countermeasures for M. Tuberculosis,” Critical Reviews™ in Eukaryotic Gene Expression 22, no. 3 (2012): 179–187. 10.1615/critreveukargeneexpr.v22.i3.10 23140159

[14] M. Karbalaei Zadeh Babaki , S. Soleimanpour , and S. A. Rezaee , “Antigen 85 Complex as a Powerful Mycobacterium Tuberculosis Immunogene: Biology, Immune‐Pathogenicity, Applications in Diagnosis, and Vaccine Design,” Microbial Pathogenesis 112 (2017): 20–29. 28942172 10.1016/j.micpath.2017.08.040

[15] K. Huygen , “The Immunodominant T‐Cell Epitopes of the Mycolyl‐Transferases of the Antigen 85 Complex of M. Tuberculosis,” Frontiers in Immunology 5 (2014): 321. 25071781 10.3389/fimmu.2014.00321 PMC4089088

[16] Y. Liang , X. Wu , J. Zhang , et al., “The Treatment of Mice Infected With Multi‐Drug‐Resistant Mycobacterium Tuberculosis Using DNA Vaccines or in Combination With Rifampin,” Vaccine 26, no. 35 (2008): 4536–4540. 18602439 10.1016/j.vaccine.2008.06.066

[17] Y. Liang , X. Wu , J. Zhang , et al., “Immunogenicity and Therapeutic Effects of Ag85A/B Chimeric DNA Vaccine in Mice Infected With Mycobacterium Tuberculosis,” FEMS Immunology & Medical Microbiology 66, no. 3 (2012): 419–426. 23163873 10.1111/1574-695X.12008

[18] Y. Liang , X. Bai , J. Zhang , et al., “Ag85A/ESAT‐6 Chimeric DNA Vaccine Induces an Adverse Response in Tuberculosis‐Infected Mice,” Molecular Medicine Reports 14, no. 2 (2016): 1146–1152. 27279275 10.3892/mmr.2016.5364 PMC4940052

[19] Y. Liang , J. Zhang , Y. Yang , et al., “Immunogenicity and Therapeutic Effects of Recombinant Ag85AB Fusion Protein Vaccines in Mice Infected With Mycobacterium Tuberculosis,” Vaccine 35, no. 32 (2017): 3995–4001. 28625522 10.1016/j.vaccine.2017.05.083

[20] Y. Liang , L. Cui , L. Xiao , et al., “Immunotherapeutic Effects of Different Doses of Mycobacterium Tuberculosis ag85a/b DNA Vaccine Delivered by Electroporation,” Frontiers in Immunology 13 (2022): 876579. 35603155 10.3389/fimmu.2022.876579 PMC9114437

[21] C. Wang , R. Fu , Z. Chen , et al., “Immunogenicity and Protective Efficacy of a Novel Recombinant BCG Strain Overexpressing Antigens Ag85A and Ag85B,” Clinical & Developmental Immunology 2012 (2012): 563838. 22570667 10.1155/2012/563838 PMC3337592

[22] W. Li , G. Deng , M. Li , et al., “A Recombinant Adenovirus Expressing CFP10, ESAT6, Ag85A and Ag85B of Mycobacterium Tuberculosis Elicits Strong Antigen‐Specific Immune Responses in Mice,” Molecular Immunology 62, no. 1 (2014): 86–95. 24980867 10.1016/j.molimm.2014.06.007

[23] S. K. Gupta , T. Wilson , P. H. Maclean , et al., “Mycobacterium Avium Subsp. Paratuberculosis Antigens Induce Cellular Immune Responses in Cattle Without Causing Reactivity to Tuberculin in the Tuberculosis Skin Test,” Frontiers in Immunology 13 (2022): 1087015. 36741398 10.3389/fimmu.2022.1087015 PMC9889921

[24] D. Sivakumaran , G. Blatner , R. Bakken , et al., “A 2‐Dose AERAS‐402 Regimen Boosts CD8(+) Polyfunctionality in HIV‐Negative, BCG‐Vaccinated Recipients,” Frontiers in Immunology 12 (2021): 673532. 34177914 10.3389/fimmu.2021.673532 PMC8231292

[25] E. Nemes , A. C. Hesseling , M. Tameris , et al., “Safety and Immunogenicity of Newborn MVA85A Vaccination and Selective, Delayed Bacille Calmette‐Guerin for Infants of Human Immunodeficiency Virus‐Infected Mothers: A Phase 2 Randomized, Controlled Trial,” Clinical Infectious Diseases 66, no. 4 (2018): 554–563. 29028973 10.1093/cid/cix834 PMC5849090

[26] F. Smaill and Z. Xing , “Human Type 5 Adenovirus‐Based Tuberculosis Vaccine: Is the Respiratory Route of Delivery the Future,” Expert Review of Vaccines 13, no. 8 (2014): 927–930. 24935214 10.1586/14760584.2014.929947

[27] A. Wajja , B. Nassanga , A. Natukunda , et al., “Safety and Immunogenicity of ChAdOx1 85A Prime Followed by MVA85A Boost Compared With BCG Revaccination Among Ugandan Adolescents Who Received BCG at Birth: A Randomised, Open‐Label Trial,” Lancet Infectious Diseases 24, no. 8 (2023): 285–296.38012890 10.1016/S1473-3099(23)00501-7

[28] A. P. Shurygina , N. Zabolotnykh , T. Vinogradova , et al., “Preclinical Evaluation of TB/FLU‐04L‐An Intranasal Influenza Vector‐Based Boost Vaccine Against Tuberculosis,” International Journal of Molecular Sciences 24, no. 8 (2023): 7439. 37108602 10.3390/ijms24087439 PMC10138401

[29] A. P. Tkachuk , E. N. Bykonia , L. I. Popova , et al., “Safety and Immunogenicity of the GamTBvac, the Recombinant Subunit Tuberculosis Vaccine Candidate: A Phase II, Multi‐Center, Double‐Blind, Randomized, Placebo‐Controlled Study,” Vaccines 8, no. 4 (2020): 652. 33153191 10.3390/vaccines8040652 PMC7712213

[30] J. Hussein , M. Zewdie , L. Yamuah , et al., “A Phase I, Open‐Label Trial on the Safety and Immunogenicity of the Adjuvanted Tuberculosis Subunit Vaccine H1/IC31® in People Living in a TB‐Endemic Area,” Trials 19, no. 1 (2018): 24. 29321075 10.1186/s13063-017-2354-0 PMC5764015

[31] L. G. Bekker , O. Dintwe , A. Fiore‐Gartland , et al., “A Phase 1b Randomized Study of the Safety and Immunological Responses to Vaccination With H4:IC31, H56:IC31, and BCG Revaccination in Mycobacterium Tuberculosis‐Uninfected Adolescents in Cape Town, South Africa,” EClinicalMedicine 21 (2020): 100313. 32382714 10.1016/j.eclinm.2020.100313 PMC7201034

[32] X. Guo , J. Lu , J. Li , et al., “The Subunit AEC/BC02 Vaccine Combined With Antibiotics Provides Protection in Mycobacterium Tuberculosis‐Infected Guinea Pigs,” Vaccines 10, no. 12 (2022): 2164. 36560574 10.3390/vaccines10122164 PMC9781032

[33] P. R. Jungblut , U. E. Schaible , H. J. Mollenkopf , et al., “Comparative Proteome Analysis of Mycobacterium Tuberculosis and Mycobacterium Bovis BCG Strains: Towards Functional Genomics of Microbial Pathogens,” Molecular Microbiology 33, no. 6 (1999): 1103–1117. 10510226 10.1046/j.1365-2958.1999.01549.x

[34] K. J. Downing , J. C. Betts , D. I. Young , et al., “Global Expression Profiling of Strains Harbouring Null Mutations Reveals That the Five RPF‐Like Genes of Mycobacterium Tuberculosis Show Functional Redundancy,” Tuberculosis 84, no. 3–4 (2004): 167–179. 15207486 10.1016/j.tube.2003.12.004

[35] J. Mattow , P. R. Jungblut , U. E. Schaible , et al., “Identification of Proteins from Mycobacterium Tuberculosis Missing in Attenuated Mycobacterium Bovis BCG Strains,” Electrophoresis 22, no. 14 (2001): 2936–2946. 11565788 10.1002/1522-2683(200108)22:14<2936::AID-ELPS2936>3.0.CO;2-S

[36] S. D. Schuck , H. Mueller , F. Kunitz , et al., “Identification of T‐Cell Antigens Specific for Latent Mycobacterium Tuberculosis Infection,” PLoS One 4, no. 5 (2009): e5590. 19440342 10.1371/journal.pone.0005590 PMC2680040

[37] S. T. Reece , A. Nasser‐Eddine , J. Dietrich , et al., “Improved Long‐Term Protection against Mycobacterium Tuberculosis Beijing/W in Mice After Intra‐Dermal Inoculation of Recombinant BCG Expressing Latency Associated Antigens,” Vaccine 29, no. 47 (2011): 8740–8744. 21871515 10.1016/j.vaccine.2011.07.144

[38] Y. Liang , Y. Zhao , X. Bai , et al., “Immunotherapeutic Effects of Mycobacterium Tuberculosis Rv3407 DNA Vaccine in Mice,” Autoimmunity 51, no. 8 (2018): 417–422. 30632804 10.1080/08916934.2018.1546291

[39] Y. Liang , X. Li , Y. Yang , et al., “Preventive Effects of Mycobacterium Tuberculosis DNA Vaccines on the Mouse Model With Latent Tuberculosis Infection,” Frontiers in Immunology 14 (2023): 1110843. 36860878 10.3389/fimmu.2023.1110843 PMC9968874

[40] M. Coppola , S. J. F. van den Eeden , L. Wilson , K. L. M. C. Franken , T. H. M. Ottenhoff , and A. Geluk , “Synthetic Long Peptide Derived From Mycobacterium Tuberculosis Latency Antigen Rv1733c Protects against Tuberculosis,” Clinical and Vaccine Immunology 22, no. 9 (2015): 1060–1069. 26202436 10.1128/CVI.00271-15 PMC4550671

[41] W. Zhang , H. Jiang , Y. Bai , J. Kang , Z. K. Xu , and L. M. Wang , “Construction and Immunogenicity of the DNA Vaccine of Mycobacterium Tuberculosis Dormancy Antigen Rv1733c,” Scandinavian Journal of Immunology 79, no. 5 (2014): 292–298. 24498941 10.1111/sji.12160

[42] A. S. De Groot , L. Moise , F. Terry , et al., “Better Epitope Discovery, Precision Immune Engineering, and Accelerated Vaccine Design Using Immunoinformatics Tools,” Frontiers in Immunology 11 (2020): 442. 32318055 10.3389/fimmu.2020.00442 PMC7154102

[43] W. Fleri , S. Paul , S. K. Dhanda , et al., “The Immune Epitope Database and Analysis Resource in Epitope Discovery and Synthetic Vaccine Design,” Frontiers In Immunology 8 (2017): 278. 28352270 10.3389/fimmu.2017.00278 PMC5348633

[44] A. N. Oli , W. O. Obialor , M. O. Ifeanyichukwu , et al., “Immunoinformatics and Vaccine Development: An Overview,” ImmunoTargets and Therapy 9 (2020): 13–30. 32161726 10.2147/ITT.S241064 PMC7049754

[45] V. H. Urrutia‐Baca , R. Gomez‐Flores , M. A. De La Garza‐Ramos , P. Tamez‐Guerra , D. G. Lucio‐Sauceda , and M. C. Rodríguez‐Padilla , “Immunoinformatics Approach to Design a Novel Epitope‐Based Oral Vaccine Against Helicobacter Pylori,” Journal of Computational Biology 26, no. 10 (2019): 1177–1190. 31120321 10.1089/cmb.2019.0062 PMC6786345

[46] I. Dimitrov , I. Bangov , D. R. Flower , and I. Doytchinova , “AllerTOP v.2–A Server for in Silico Prediction of Allergens,” Journal of Molecular Modeling 20, no. 6 (2014): 2278. 24878803 10.1007/s00894-014-2278-5

[47] T. N. Petersen , S. Brunak , G. von Heijne , and H. Nielsen , “Signalp 4.0: Discriminating Signal Peptides From Transmembrane Regions,” Nature Methods 8, no. 10 (2011): 785–786. 21959131 10.1038/nmeth.1701

[48] H. B. Shen and K. C. Chou , “A Top‐Down Approach to Enhance the Power of Predicting Human Protein Subcellular Localization: Hum‐mPLoc 2.0,” Analytical Biochemistry 394, no. 2 (2009): 269–274. 19651102 10.1016/j.ab.2009.07.046

[49] I. A. Doytchinova and D. R. Flower , “Vaxijen: A Server for Prediction of Protective Antigens, Tumour Antigens and Subunit Vaccines,” BMC Bioinformatics 8 (2007): 4. 17207271 10.1186/1471-2105-8-4 PMC1780059

[50] S. Gupta , P. Kapoor , K. Chaudhary , A. Gautam , R. Kumar , and G. P. S. Raghava , “In Silico Approach for Predicting Toxicity of Peptides and Proteins,” PLoS One 8, no. 9 (2013): e73957. 24058508 10.1371/journal.pone.0073957 PMC3772798

[51] J. Ye , S. McGinnis , and T. L. Madden , “Blast: Improvements for Better Sequence Analysis,” Nucleic Acids Research 34, no. Web Server issue (2006): W6–W9. 16845079 10.1093/nar/gkl164 PMC1538791

[52] C. Geourjon and G. Deléage , “SOPMA: Significant Improvements in Protein Secondary Structure Prediction by Consensus Prediction From Multiple Alignments,” Bioinformatics 11, no. 6 (1995): 681–684. 10.1093/bioinformatics/11.6.681 8808585

[53] J. Jumper , R. Evans , A. Pritzel , et al., “Highly Accurate Protein Structure Prediction With Alphafold,” Nature 596, no. 7873 (2021): 583–589. 34265844 10.1038/s41586-021-03819-2 PMC8371605

[54] M. Wiederstein and M. J. Sippl , “ProSA‐Web: Interactive Web Service for the Recognition of Errors in Three‐Dimensional Structures of Proteins,” Nucleic Acids Research 35, no. Web Server issue (2007): W407–W410. 17517781 10.1093/nar/gkm290 PMC1933241

[55] I. A. Vakser , “Long‐Distance Potentials: An Approach to the Multiple‐Minima Problem in Ligand‐Receptor Interaction,” "Protein Engineering, Design and Selection" 9, no. 1 (1996): 37–41. 10.1093/protein/9.1.37 9053900

[56] S. K. Burley , H. M. Berman , C. Bhikadiya , et al., “Protein Data Bank: The Single Global Archive for 3D Macromolecular Structure Data,” Nucleic Acids Research 47, no. D1 (2019): D520–D528. 30357364 10.1093/nar/gky949 PMC6324056

[57] E. Krissinel and K. Henrick , “Inference of Macromolecular Assemblies From Crystalline State,” Journal of Molecular Biology 372, no. 3 (2007): 774–797. 17681537 10.1016/j.jmb.2007.05.022

[58] J. Ponomarenko , H. H. Bui , W. Li , et al., “Ellipro: A New Structure‐Based Tool for the Prediction of Antibody Epitopes,” BMC Bioinformatics 9 (2008): 514. 19055730 10.1186/1471-2105-9-514 PMC2607291

[59] P. Wang , J. Sidney , Y. Kim , et al., “Peptide Binding Predictions for HLA DR, DP and DQ Molecules,” BMC Bioinformatics 11 (2010): 568. 21092157 10.1186/1471-2105-11-568 PMC2998531

[60] S. K. Dhanda , P. Vir , and G. P. Raghava , “Designing of Interferon‐Gamma Inducing MHC Class‐II Binders,” Biology Direct 8 (2013): 30. 24304645 10.1186/1745-6150-8-30 PMC4235049

[61] S. K. Dhanda , S. Gupta , P. Vir , and G. P. Raghava , “Prediction of IL4 Inducing Peptides,” Clinical & Developmental Immunology 2013 (2013): 263952. 24489573 10.1155/2013/263952 PMC3893860

[62] A. Dhall , S. Patiyal , N. Sharma , S. S. Usmani , and G. P. S. Raghava , “Computer‐Aided Prediction and Design of IL‐6 Inducing Peptides: IL‐6 Plays a Crucial Role in Covid‐19,” Briefings in Bioinformatics 22, no. 2 (2021): 936–945. 33034338 10.1093/bib/bbaa259 PMC7665369

[63] G. Nagpal , S. S. Usmani , S. K. Dhanda , et al., “Computer‐Aided Designing of Immunosuppressive Peptides Based on IL‐10 Inducing Potential,” Scientific Reports 7 (2017): 42851. 28211521 10.1038/srep42851 PMC5314457

[64] M. Andreatta and M. Nielsen , “Gapped Sequence Alignment Using Artificial Neural Networks: Application to the Mhc Class I System,” Bioinformatics 32, no. 4 (2016): 511–517. 26515819 10.1093/bioinformatics/btv639 PMC6402319

[65] J. J. A. Calis , M. Maybeno , J. A. Greenbaum , et al., “Properties of MHC Class I Presented Peptides That Enhance Immunogenicity,” PLoS Computational Biology 9, no. 10 (2013): e1003266. 24204222 10.1371/journal.pcbi.1003266 PMC3808449

[66] H. H. Bui , J. Sidney , K. Dinh , S. Southwood , M. J. Newman , and A. Sette , “Predicting Population Coverage of T‐Cell Epitope‐Based Diagnostics and Vaccines,” BMC Bioinformatics 7 (2006): 153. 16545123 10.1186/1471-2105-7-153 PMC1513259

[67] C. Alland , F. Moreews , D. Boens , et al., “Rpbs: A Web Resource for Structural Bioinformatics,” Nucleic Acids Research 33, no. Web Server issue (2005): W44–W49. 15980507 10.1093/nar/gki477 PMC1160237

[68] N. Rapin , O. Lund , M. Bernaschi , and F. Castiglione , “Computational Immunology Meets Bioinformatics: The Use of Prediction Tools for Molecular Binding in the Simulation of the Immune System,” PLoS One 5, no. 4 (2010): e9862. 20419125 10.1371/journal.pone.0009862 PMC2855701

[69] R. Michael and J. S. Green , Molecular Cloning: A Laboratory (Manual Science Press Co. Ltd., 2017).

[70] R. Michael and J. S. Green Molecular Cloning: A Laboratory Manual (Fourth Edition): Three‐Volume Set. 2012.

[71] A. Moodley , A. Fatoba , M. Okpeku , T. Emmanuel Chiliza , M. Blessing Cedric Simelane , and O. J. Pooe , “Reverse Vaccinology Approach to Design a Multi‐Epitope Vaccine Construct Based on the Mycobacterium Tuberculosis Biomarker Pe_Pgrs17,” Immunologic Research 70, no. 4 (2022): 501–517. 35554858 10.1007/s12026-022-09284-x PMC9095442

[72] S. Scheiblhofer , J. Laimer , Y. Machado , R. Weiss , and J. Thalhamer , “Influence of Protein Fold Stability on Immunogenicity and Its Implications for Vaccine Design,” Expert Review of Vaccines 16, no. 5 (2017): 479–489. 28290225 10.1080/14760584.2017.1306441 PMC5490637

[73] K. Saylor , F. Gillam , T. Lohneis , and C. Zhang , “Designs of Antigen Structure and Composition for Improved Protein‐Based Vaccine Efficacy,” Frontiers in immunology 11 (2020): 283. 32153587 10.3389/fimmu.2020.00283 PMC7050619

[74] A. Kazakova , P. Zhelnov , R. Sidorov , et al., “DNA and RNA Vaccines Against Tuberculosis: A Scoping Review of Human and Animal Studies,” Frontiers in Immunology 15 (2024): 1457327. 39421744 10.3389/fimmu.2024.1457327 PMC11483866

[75] P. Stewart , S. Patel , A. Comer , et al., “Role of B Cells in Mycobacterium Tuberculosis Infection,” Vaccines 11, no. 5 (2023): 955. 37243059 10.3390/vaccines11050955 PMC10222439

[76] D. Accapezzato , V. Visco , V. Francavilla , et al., “Chloroquine Enhances Human CD8+ T Cell Responses Against Soluble Antigens In Vivo,” Journal of Experimental Medicine 202, no. 6 (2005): 817–828. 16157687 10.1084/jem.20051106 PMC2212941

[77] L. Delamarre , M. Pack , H. Chang , I. Mellman , and E. S. Trombetta , “Differential Lysosomal Proteolysis in Antigen‐Presenting Cells Determines Antigen Fate,” Science 307, no. 5715 (2005): 1630–1634. 15761154 10.1126/science.1108003

[78] L. Delamarre , R. Couture , I. Mellman , and E. S. Trombetta , “Enhancing Immunogenicity by Limiting Susceptibility to Lysosomal Proteolysis,” Journal of Experimental Medicine 203, no. 9 (2006): 2049–2055. 16908625 10.1084/jem.20052442 PMC2118388

[79] M. S. Rodriguez , J. M. P. Desterro , S. Lain , D. P. Lane , and R. T. Hay , “Multiple C‐Terminal Lysine Residues Target p53 for Ubiquitin‐Proteasome‐Mediated Degradation,” Molecular and Cellular Biology 20, no. 22 (2000): 8458–8467. 11046142 10.1128/mcb.20.22.8458-8467.2000 PMC102152

[80] B. Khamsri , M. Fujita , K. Kamada , et al., “Effects of Lysine to Arginine Mutations in HIV‐1 Vif on Its Expression and Viral Infectivity,” International Journal of Molecular Medicine 18, no. 4 (2006): 679–683. 16964423

[81] R. T. Timms , Z. Zhang , D. Y. Rhee , J. W. Harper , I. Koren , and S. J. Elledge , “A Glycine‐Specific N‐Degron Pathway Mediates the Quality Control of Protein N‐Myristoylation,” Science 365, no. 6448 (2019): eaaw4912. 31273098 10.1126/science.aaw4912 PMC7090375

[82] M. Faridgohar and H. Nikoueinejad , “New Findings of Toll‐Like Receptors Involved in Mycobacterium Tuberculosis Infection,” Pathogens and Global Health 111, no. 5 (2017): 256–264. 28715935 10.1080/20477724.2017.1351080 PMC5560203

[83] A. Ciesielska , M. Matyjek , and K. Kwiatkowska , “TLR4 and CD14 Trafficking and its Influence on Lps‐Induced Pro‐Inflammatory Signaling,” Cellular and Molecular Life Sciences 78, no. 4 (2021): 1233–1261. 33057840 10.1007/s00018-020-03656-y PMC7904555

[84] J. Geng , Y. Shi , J. Zhang , et al., “TLR4 Signalling via Piezo1 Engages and Enhances the Macrophage Mediated Host Response during Bacterial Infection,” Nature Communications 12, no. 1 (2021): 3519. 10.1038/s41467-021-23683-y PMC8192512 34112781

[85] C. Saelee , J. Hanthamrongwit , P. T. Soe , et al., “Toll‐Like Receptor‐Mediated Innate Immune Responses by Recognition of the Recombinant Dormancy‐Associated Mycobacterium Tuberculosis Proteins Rv2659c and Rv1738,” PLoS One 17, no. 9 (2022): e0273517. 36048884 10.1371/journal.pone.0273517 PMC9436120

[86] S. Pahari , S. Negi , M. Aqdas , E. Arnett , L. S. Schlesinger , and J. N. Agrewala , “Induction of Autophagy through CLEC4E in Combination With TLR4: An Innovative Strategy to Restrict the Survival of Mycobacterium Tuberculosis,” Autophagy 16, no. 6 (2020): 1021–1043. 31462144 10.1080/15548627.2019.1658436 PMC7469444

[87] E. K. Jo , J. K. Park , and H. M. Dockrell , “Dynamics of Cytokine Generation in Patients With Active Pulmonary Tuberculosis,” Current Opinion in Infectious Diseases 16, no. 3 (2003): 205–210. 12821809 10.1097/00001432-200306000-00004

[88] C. Lienhardt , A. Azzurri , A. Amedei , et al., “Active Tuberculosis in Africa Is Associated With Reduced Th1 and Increased Th2 Activity in Vivo,” European Journal of Immunology 32, no. 6 (2002): 1605–1613. 12115643 10.1002/1521-4141(200206)32:6<1605::AID-IMMU1605>3.0.CO;2-6

[89] T. J. Scriba , M. G. Netea , and A. M. Ginsberg , “Key Recent Advances in TB Vaccine Development and Understanding of Protective Immune Responses Against Mycobacterium Tuberculosis,” Seminars in Immunology 50 (2020): 101431. 33279383 10.1016/j.smim.2020.101431 PMC7786643

[90] M. Saqib , R. Khatri , B. Singh , A. Gupta , A. Kumar , and S. Bhaskar , “Mycobacterium Indicus Pranii as a Booster Vaccine Enhances BCG Induced Immunity and Confers Higher Protection in Animal Models of Tuberculosis,” Tuberculosis 101 (2016): 164–173. 27865389 10.1016/j.tube.2016.10.002

[91] R. Biselli , S. Mariotti , V. Sargentini , et al., “Detection of interleukin‐2 in Addition to interferon‐γ Discriminates Active Tuberculosis Patients, Latently Infected Individuals, and Controls,” Clinical Microbiology and Infection 16, no. 8 (2010): 1282–1284. 19886902 10.1111/j.1469-0691.2009.03104.x

[92] A. Penn‐Nicholson , M. Tameris , E. Smit , et al., “Safety and Immunogenicity of the Novel Tuberculosis Vaccine ID93 + Gla‐Se in BCG‐Vaccinated Healthy Adults in South Africa: A Randomised, Double‐Blind, Placebo‐Controlled Phase 1 Trial,” Lancet Respiratory Medicine 6, no. 4 (2018): 287–298. 29595510 10.1016/S2213-2600(18)30077-8

[93] P. Ogongo , L. B. Tezera , A. Ardain , et al., “Tissue‐Resident‐Like CD4+ T Cells Secreting IL‐17 Control Mycobacterium Tuberculosis in the Human Lung,” Journal of Clinical Investigation 131, no. 10 (2021): e142014. 33848273 10.1172/JCI142014 PMC8121523

[94] J. A. Wik and B. S. Skålhegg , “T Cell Metabolism in Infection,” Frontiers in Immunology 13 (2022): 840610. 35359994 10.3389/fimmu.2022.840610 PMC8964062

[95] N. V. Serbina , V. Lazarevic , and J. L. Flynn , “CD4(+) T Cells are Required for the Development of Cytotoxic CD8(+) T Cells During Mycobacterium Tuberculosis Infection,” Journal of Immunology 167, no. 12 (2001): 6991–7000. 10.4049/jimmunol.167.12.6991 11739519

[96] M. Okada , Y. Kita , T. Nakajima , et al., “The Study of Novel DNA Vaccines Against Tuberculosis: Induction of Pathogen‐Specific CTL in the Mouse and Monkey Models of Tuberculosis,” Human Vaccines & Immunotherapeutics 9, no. 3 (2013): 515–525. 23249543 10.4161/hv.23229 PMC3891707

[97] M. H. Lew , M. N. Norazmi , F. Nordin , and G. J. Tye , “A Novel Peptide Vaccination Augments Cytotoxic CD8(+) T‐Cell Responses against Mycobacterium Tuberculosis HSPX Antigen,” Immunobiology 227, no. 3 (2022): 152201. 35272134 10.1016/j.imbio.2022.152201

[98] S. M. Smith , R. Brookes , R. Klein , et al., “Human CD8+ CTL Specific for the Mycobacterial Major Secreted Antigen 85A,” Journal of Immunology 165, no. 12 (2000): 7088–7095. 10.4049/jimmunol.165.12.7088 11120838

[99] P. Launois , R. DeLeys , M. N. Niang , et al., “T‐Cell‐Epitope Mapping of the Major Secreted Mycobacterial Antigen Ag85A in Tuberculosis and Leprosy,” Infection and Immunity 62, no. 9 (1994): 3679–3687. 7520418 10.1128/iai.62.9.3679-3687.1994 PMC303018

