# Supplementary material for: Bioinformatics Analysis and Immunogenicity Assessment of the Novel Multi‐Stage DNA Vaccine W541 Against Mycobacterium Tuberculosis
Source: Immun Inflamm Dis. 2024 Nov 26;12(11):e70074. doi: 10.1002/iid3.70074 (PMC11590035; doi:10.1002/iid3.70074)
Supplement: Supplementary file 1 — Supporting information. [file IID3-12-e70074-s004.docx]

Supplementary Table 1A B-cell linear epitopes of W541 vaccine protein

| No. | Source | Start | End | Peptides | Number of residues | Score |
| --- | --- | --- | --- | --- | --- | --- |
| 1 | Ag85B | 434 | 450 | SELPQWLSANRAVKPTG | 17 | 0.755 |
| 2 | Ag85A | 1 | 35 | FSRPGLPVEYLQVPSPSMGRDIKVQFQSGGANSPA | 35 | 0.731 |
| 3 | Ag85B | 327 | 366 | ATAGAFSRPGLPVEYLQVPSPSMGRDIKVQFQSGGNNSPA | 40 | 0.711 |
| 4 | Ag85A | 89 | 120 | KAGCQTYKWETFLTSELPGWLQANRHVKPTGS | 32 | 0.676 |
| 5 | Ag85A | 62 | 69 | DQSGLSVV | 8 | 0.652 |
| 6 | Ag85A | 245 | 251 | NAGGGHN | 7 | 0.638 |
| 7 | Ag85A | 138 | 144 | YHPQQFV | 7 | 0.607 |
| 8 | Ag85B | 386 | 400 | TPAFEWYYQSGLSIV | 15 | 0.599 |
| 9 | Ag85A | 197 | 205 | VGKLIANNT | 9 | 0.587 |
| 10 | Ag85B | 420 | 427 | KAGCQTYK | 8 | 0.562 |
| 11 | Ag85B | 470 | 475 | HPQQFI | 6 | 0.548 |
| 12 | Ag85B | 577 | 582 | AAGGHN | 6 | 0.546 |
| 13 | Ag85A | 80 | 87 | SDWYQPAC | 8 | 0.542 |
| 14 | Ag85B | 532 | 536 | VANNT | 5 | 0.531 |
| 15 | Ag85B | 411 | 418 | SDWYSPAC | 8 | 0.511 |

Supplementary Table 1B B-cell discontinuous epitopes of W541 vaccine protein

| No. | Residues | Number of residues | Score |
| --- | --- | --- | --- |
| 1 | Q698, A699, Q700, T701, R702 | 5 | 0.987 |
| 2 | V694, Y695, A696, H697 | 4 | 0.979 |
| 3 | R644, P645, Q646, N647, L648, L649, D650, T652 | 8 | 0.908 |
| 4 | I308, G309, T310, A311, A312, A313, V314 | 7 | 0.903 |
| 5 | A653, E654, P655, A656, R657 | 5 | 0.893 |
| 6 | G658, R659, K660 | 3 | 0.836 |
| 7 | V315, L316, P317, G318, L319, V320, G321 | 7 | 0.724 |
| 8 | R661, T662, S664, D665, N668, R671 | 6 | 0.687 |
| 9 | Y83, Q84, P85, C87, A90, G91, C92, Q93, Y95 | 9 | 0.59 |
| 10 | W413, S415, P416, C418, A421, G422, C423, Q424, Y426 | 9 | 0.554 |
| 11 | P529, V532, N535, A577, A578, G579, G580, H581, N582 | 9 | 0.543 |
