# Supplementary material for: Bioinformatics Analysis and Immunogenicity Assessment of the Novel Multi‐Stage DNA Vaccine W541 Against Mycobacterium Tuberculosis
Source: Immun Inflamm Dis. 2024 Nov 26;12(11):e70074. doi: 10.1002/iid3.70074 (PMC11590035; doi:10.1002/iid3.70074)
Supplement: Supplementary file 2 — Supporting information. [file IID3-12-e70074-s002.docx]

**Supplementary Table 2 Predicted HTL epitopes of W541 vaccine protein**

| allele | peptide | Source | start | end | antigenicity | IFN induce | IL4 induce | IL6 induce | IL10 induce |
| --- | --- | --- | --- | --- | --- | --- | --- | --- | --- |
| HLA-DPA1*03:01/DPB1*04:02,HLA-DPA1*03:01/DPB1*04:02,HLA-DRB4*01:01,HLA-DRB4*01:01,HLA-DPA1*01:03/DPB1*02:01,HLA-DPA1*01:03/DPB1*02:01 | SRPGLPVEYLQVPSP | Ag85A | 2 | 16 | 0.443 | + | - | - | - |
| HLA-DPA1*03:01/DPB1*04:02,HLA-DPA1*03:01/DPB1*04:02,HLA-DRB4*01:01,HLA-DRB4*01:01,HLA-DPA1*01:03/DPB1*02:01,HLA-DPA1*01:03/DPB1*02:01,HLA-DRB1*04:05,HLA-DRB1*04:05 | RPGLPVEYLQVPSPS | Ag85A | 3 | 17 | 0.6066 | + | - | - | - |
| HLA-DRB1*04:01,HLA-DRB1*04:01,HLA-DRB1*04:05,HLA-DRB1*04:05,HLA-DRB1*09:01,HLA-DRB1*09:01,HLA-DRB1*07:01,HLA-DRB1*07:01,HLA-DRB4*01:01,HLA-DRB4*01:01,HLA-DPA1*03:01/DPB1*04:02,HLA-DPA1*03:01/DPB1*04:02,HLA-DRB1*01:01,HLA-DRB1*01:01,HLA-DRB1*11:01,HLA-DRB1*11:01,HLA-DPA1*01:03/DPB1*02:01,HLA-DPA1*01:03/DPB1*02:01,HLA-DRB5*01:01,HLA-DRB5*01:01,HLA-DRB3*01:01,HLA-DRB3*01:01 | PGLPVEYLQVPSPSM | Ag85A | 4 | 18 | 0.7752 | + | - | - | - |
| HLA-DRB1*04:01,HLA-DRB1*04:01,HLA-DRB1*01:01,HLA-DRB1*01:01,HLA-DRB1*04:05,HLA-DRB1*04:05,HLA-DRB1*09:01,HLA-DRB1*09:01,HLA-DRB1*08:02,HLA-DRB1*08:02,HLA-DRB1*07:01,HLA-DRB1*07:01,HLA-DRB4*01:01,HLA-DRB4*01:01,HLA-DRB5*01:01,HLA-DRB5*01:01,HLA-DRB1*11:01,HLA-DRB1*11:01,HLA-DRB3*01:01,HLA-DRB3*01:01 | GLPVEYLQVPSPSMG | Ag85A | 5 | 19 | 0.7985 | + | - | - | - |
| HLA-DRB1*04:01,HLA-DRB1*04:01,HLA-DRB1*01:01,HLA-DRB1*01:01,HLA-DRB3*02:02,HLA-DRB3*02:02,HLA-DRB1*04:05,HLA-DRB1*04:05,HLA-DRB1*09:01,HLA-DRB1*09:01,HLA-DRB5*01:01,HLA-DRB5*01:01,HLA-DRB1*07:01,HLA-DRB1*07:01,HLA-DRB1*08:02,HLA-DRB1*08:02,HLA-DRB4*01:01,HLA-DRB4*01:01,HLA-DRB1*11:01,HLA-DRB1*11:01,HLA-DRB1*15:01,HLA-DRB1*15:01,HLA-DRB3*01:01,HLA-DRB3*01:01 | LPVEYLQVPSPSMGR | Ag85A | 6 | 20 | 0.7922 | + | - | - | - |
| HLA-DRB1*04:01,HLA-DRB1*04:01,HLA-DRB1*01:01,HLA-DRB1*01:01,HLA-DRB3*02:02,HLA-DRB3*02:02,HLA-DRB1*04:05,HLA-DRB1*04:05,HLA-DRB1*09:01,HLA-DRB1*09:01,HLA-DRB5*01:01,HLA-DRB5*01:01,HLA-DRB1*07:01,HLA-DRB1*07:01,HLA-DRB1*11:01,HLA-DRB1*11:01,HLA-DRB1*08:02,HLA-DRB1*08:02,HLA-DRB4*01:01,HLA-DRB4*01:01,HLA-DRB3*01:01,HLA-DRB3*01:01 | PVEYLQVPSPSMGRD | Ag85A | 7 | 21 | 0.7094 | + | - | - | - |
| HLA-DRB1*04:01,HLA-DRB1*04:01,HLA-DRB1*01:01,HLA-DRB1*01:01,HLA-DRB1*04:05,HLA-DRB1*04:05,HLA-DRB1*09:01,HLA-DRB1*09:01,HLA-DRB5*01:01,HLA-DRB5*01:01,HLA-DRB1*07:01,HLA-DRB1*07:01,HLA-DRB1*11:01,HLA-DRB1*11:01,HLA-DRB1*08:02,HLA-DRB1*08:02,HLA-DRB3*01:01,HLA-DRB3*01:01 | VEYLQVPSPSMGRDI | Ag85A | 8 | 22 | 0.7257 | + | - | - | - |
| HLA-DRB1*04:01,HLA-DRB1*04:01,HLA-DRB1*01:01,HLA-DRB1*01:01,HLA-DRB1*04:05,HLA-DRB1*04:05,HLA-DRB1*07:01,HLA-DRB1*07:01,HLA-DRB1*09:01,HLA-DRB1*09:01,HLA-DRB5*01:01,HLA-DRB5*01:01,HLA-DRB3*01:01,HLA-DRB3*01:01 | EYLQVPSPSMGRDIK | Ag85A | 9 | 23 | 0.9509 | + | - | - | - |
| HLA-DRB1*04:01,HLA-DRB1*04:01,HLA-DRB1*01:01,HLA-DRB1*01:01,HLA-DRB1*07:01,HLA-DRB1*07:01,HLA-DRB5*01:01,HLA-DRB5*01:01 | YLQVPSPSMGRDIKV | Ag85A | 10 | 24 | 1.5272 | + | - | - | - |
| HLA-DRB1*03:01,HLA-DRB1*03:01,HLA-DRB4*01:01,HLA-DRB4*01:01 | QVPSPSMGRDIKVQF | Ag85A | 12 | 26 | 1.595 | + | - | - | - |
| HLA-DRB1*03:01,HLA-DRB1*03:01,HLA-DRB4*01:01,HLA-DRB4*01:01 | VPSPSMGRDIKVQFQ | Ag85A | 13 | 27 | 1.6767 | + | - | - | - |
| HLA-DRB1*03:01,HLA-DRB1*03:01,HLA-DRB4*01:01,HLA-DRB4*01:01,HLA-DQA1*01:02/DQB1*06:02,HLA-DQA1*01:02/DQB1*06:02 | PSPSMGRDIKVQFQS | Ag85A | 14 | 28 | 1.483 | + | - | - | - |
| HLA-DRB1*04:01,HLA-DQA1*05:01/DQB1*03:01,HLA-DRB4*01:01,HLA-DRB1*15:01,HLA-DRB1*01:01 | DIKVQFQSGGANSPA | Ag85A | 21 | 35 | 1.5849 | + | - | - | - |
| HLA-DRB1*04:01,HLA-DQA1*05:01/DQB1*03:01,HLA-DRB1*01:01,HLA-DRB4*01:01 | IKVQFQSGGANSPAL | Ag85A | 22 | 36 | 1.4355 | - | + | - | - |
| HLA-DRB3*01:01,HLA-DRB1*04:05,HLA-DRB1*01:01,HLA-DRB1*04:01,HLA-DRB4*01:01,HLA-DRB1*12:01,HLA-DPA1*03:01/DPB1*04:02 | LYLLDGLRAQDDFSG | Ag85A | 36 | 50 | 0.5076 | + | + | - | + |
| HLA-DQA1*01:01/DQB1*05:01 | LRAQDDFSGWDINTP | Ag85A | 42 | 56 | 0.9889 | + | + | - | + |
| HLA-DQA1*01:01/DQB1*05:01,HLA-DRB1*09:01,HLA-DPA1*02:01/DPB1*01:01 | DINTPAFEWYDQSGL | Ag85A | 52 | 66 | 0.7697 | + | + | - | - |
| HLA-DQA1*01:01/DQB1*05:01,HLA-DRB1*09:01,HLA-DPA1*02:01/DPB1*01:01 | INTPAFEWYDQSGLS | Ag85A | 53 | 67 | 0.8507 | - | + | - | - |
| HLA-DQA1*01:01/DQB1*05:01,HLA-DRB1*09:01,HLA-DRB1*01:01,HLA-DRB1*07:01,HLA-DPA1*03:01/DPB1*04:02,HLA-DPA1*02:01/DPB1*01:01,HLA-DRB1*15:01 | TPAFEWYDQSGLSVV | Ag85A | 55 | 69 | 0.6294 | + | + | - | - |
| HLA-DQA1*01:01/DQB1*05:01,HLA-DRB1*09:01,HLA-DPA1*03:01/DPB1*04:02,HLA-DRB1*01:01,HLA-DPA1*02:01/DPB1*01:01,HLA-DRB1*07:01,HLA-DQA1*05:01/DQB1*03:01,HLA-DRB1*15:01 | PAFEWYDQSGLSVVM | Ag85A | 56 | 70 | 0.6727 | + | + | - | - |
| HLA-DQA1*01:02/DQB1*06:02,HLA-DQA1*05:01/DQB1*03:01,HLA-DRB1*09:01,HLA-DRB1*07:01,HLA-DRB1*01:01 | YDQSGLSVVMPVGGQ | Ag85A | 61 | 75 | 0.5974 | - | - | - | + |
| HLA-DQA1*01:02/DQB1*06:02,HLA-DQA1*05:01/DQB1*03:01,HLA-DRB1*01:01 | QSGLSVVMPVGGQSS | Ag85A | 63 | 77 | 0.8807 | + | - | - | + |
| HLA-DQA1*05:01/DQB1*03:01,HLA-DRB1*01:01 | SGLSVVMPVGGQSSF | Ag85A | 64 | 78 | 0.4883 | + | - | - | + |
| HLA-DQA1*01:01/DQB1*05:01,HLA-DRB3*01:01,HLA-DPA1*01:03/DPB1*02:01 | SSFYSDWYQPACGKA | Ag85A | 76 | 90 | 0.5351 | + | + | - | + |
| HLA-DQA1*01:01/DQB1*05:01,HLA-DPA1*01:03/DPB1*02:01,HLA-DRB1*04:01,HLA-DRB1*01:01 | SFYSDWYQPACGKAG | Ag85A | 77 | 91 | 0.9234 | + | + | - | + |
| HLA-DQA1*01:02/DQB1*06:02,HLA-DQA1*05:01/DQB1*03:01,HLA-DRB1*08:02,HLA-DRB1*01:01,HLA-DRB1*09:01 | KPTGSAVVGLSMAAS | Ag85A | 116 | 130 | 0.6974 | + | - | - | - |
| HLA-DQA1*01:02/DQB1*06:02,HLA-DQA1*05:01/DQB1*03:01,HLA-DRB1*01:01,HLA-DRB1*08:02,HLA-DRB1*04:01,HLA-DRB1*09:01 | PTGSAVVGLSMAASS | Ag85A | 117 | 131 | 0.7231 | + | - | - | - |
| HLA-DQA1*01:02/DQB1*06:02,HLA-DQA1*05:01/DQB1*03:01,HLA-DRB1*01:01,HLA-DRB1*08:02,HLA-DRB1*04:01,HLA-DRB1*09:01 | TGSAVVGLSMAASSA | Ag85A | 118 | 132 | 0.9493 | + | - | - | - |
| HLA-DRB1*09:01,HLA-DQA1*01:02/DQB1*06:02,HLA-DQA1*05:01/DQB1*03:01,HLA-DRB1*07:01,HLA-DRB1*08:02,HLA-DRB1*01:01,HLA-DRB1*04:01,HLA-DRB1*13:02,HLA-DRB1*04:05,HLA-DRB4*01:01,HLA-DRB5*01:01 | GSAVVGLSMAASSAL | Ag85A | 119 | 133 | 0.7072 | + | - | - | - |
| HLA-DRB1*09:01,HLA-DQA1*01:02/DQB1*06:02,HLA-DQA1*05:01/DQB1*03:01,HLA-DRB1*07:01,HLA-DRB1*08:02,HLA-DRB1*01:01,HLA-DRB1*04:01,HLA-DRB1*13:02,HLA-DRB1*04:05,HLA-DRB4*01:01,HLA-DRB1*15:01,HLA-DRB5*01:01 | SAVVGLSMAASSALT | Ag85A | 120 | 134 | 0.6602 | + | - | - | - |
| HLA-DRB1*09:01,HLA-DQA1*01:02/DQB1*06:02,HLA-DQA1*05:01/DQB1*03:01,HLA-DRB1*07:01,HLA-DRB3*02:02,HLA-DRB1*08:02,HLA-DRB1*13:02,HLA-DRB1*01:01,HLA-DRB1*04:01,HLA-DRB1*15:01,HLA-DRB1*04:05,HLA-DRB4*01:01,HLA-DRB5*01:01,HLA-DPA1*01:03/DPB1*02:01,HLA-DPA1*02:01/DPB1*01:01,HLA-DPA1*03:01/DPB1*04:02 | AVVGLSMAASSALTL | Ag85A | 121 | 135 | 0.7053 | + | - | - | - |
| HLA-DRB1*09:01,HLA-DQA1*05:01/DQB1*03:01,HLA-DRB1*07:01,HLA-DQA1*01:02/DQB1*06:02,HLA-DRB3*02:02,HLA-DPA1*01:03/DPB1*02:01,HLA-DRB1*01:01,HLA-DRB1*13:02,HLA-DPA1*02:01/DPB1*01:01,HLA-DRB1*03:01,HLA-DRB1*04:01,HLA-DRB1*04:05,HLA-DRB1*15:01,HLA-DRB5*01:01,HLA-DRB4*01:01,HLA-DPA1*03:01/DPB1*04:02 | VGLSMAASSALTLAI | Ag85A | 123 | 137 | 0.8465 | + | - | - | - |
| HLA-DQA1*05:01/DQB1*03:01 | AMGDAGGYKASDMWG | Ag85A | 167 | 181 | 0.7503 | - | + | - | - |
| HLA-DQA1*05:01/DQB1*03:01 | MGDAGGYKASDMWGP | Ag85A | 168 | 182 | 0.6618 | - | + | - | - |
| HLA-DQA1*05:01/DQB1*03:01 | GDAGGYKASDMWGPK | Ag85A | 169 | 183 | 1.1476 | - | + | - | - |
| HLA-DQA1*05:01/DQB1*03:01 | RVWVYCGNGKPSDLG | Ag85A | 206 | 220 | 0.7448 | + | - | - | - |
| HLA-DQA1*05:01/DQB1*03:01 | WVYCGNGKPSDLGGN | Ag85A | 208 | 222 | 1.4613 | + | - | - | - |
| HLA-DQA1*05:01/DQB1*03:01,HLA-DRB1*01:01 | KPSDLGGNNLPAKFL | Ag85A | 215 | 229 | 1.1072 | - | + | - | - |
| HLA-DQA1*05:01/DQB1*03:01,HLA-DRB1*01:01 | PSDLGGNNLPAKFLE | Ag85A | 216 | 230 | 0.8679 | + | + | - | - |
| HLA-DRB1*01:01 | SDLGGNNLPAKFLEG | Ag85A | 217 | 231 | 1.0008 | + | + | - | - |
| HLA-DRB1*01:01 | LGGNNLPAKFLEGFV | Ag85A | 219 | 233 | 0.5004 | + | - | - | - |
| HLA-DRB4*01:01,HLA-DRB1*07:01,HLA-DRB1*08:02,HLA-DRB1*04:01,HLA-DRB1*15:01,HLA-DRB1*11:01,HLA-DRB5*01:01,HLA-DRB1*13:02,HLA-DRB1*09:01,HLA-DRB1*01:01 | FVRTSNIKFQDAYNA | Ag85A | 232 | 246 | 1.0603 | + | + | - | - |
| HLA-DRB4*01:01,HLA-DRB1*04:01,HLA-DRB1*15:01,HLA-DRB1*08:02,HLA-DRB1*01:01,HLA-DRB1*09:01 | RTSNIKFQDAYNAGG | Ag85A | 234 | 248 | 1.5116 | - | + | - | - |
| HLA-DRB1*04:01,HLA-DRB1*15:01,HLA-DRB1*08:02,HLA-DRB4*01:01,HLA-DRB1*01:01,HLA-DRB1*09:01,HLA-DQA1*05:01/DQB1*03:01 | TSNIKFQDAYNAGGG | Ag85A | 235 | 249 | 1.746 | - | + | - | - |
| HLA-DRB1*04:01,HLA-DQA1*05:01/DQB1*03:01,HLA-DRB1*01:01 | IKFQDAYNAGGGHNG | Ag85A | 238 | 252 | 2.0509 | - | + | - | - |
| HLA-DQA1*05:01/DQB1*03:01,HLA-DRB1*01:01 | KFQDAYNAGGGHNGV | Ag85A | 239 | 253 | 1.8289 | - | + | - | - |
| HLA-DQA1*05:01/DQB1*03:01 | AYNAGGGHNGVFDFP | Ag85A | 243 | 257 | 1.3298 | - | + | - | - |
| HLA-DQA1*01:01/DQB1*05:01,HLA-DPA1*01:03/DPB1*02:01,HLA-DRB1*09:01 | SGTHSWEYWGAQLNA | Ag85A | 259 | 273 | 0.7586 | - | + | - | - |
| HLA-DQA1*01:01/DQB1*05:01,HLA-DQA1*01:01/DQB1*05:01,HLA-DPA1*01:03/DPB1*04:01,HLA-DPA1*01:03/DPB1*04:01,HLA-DPA1*02:01/DPB1*01:01,HLA-DPA1*02:01/DPB1*01:01,HLA-DPA1*01:03/DPB1*02:01,HLA-DPA1*01:03/DPB1*02:01,HLA-DQA1*05:01/DQB1*02:01,HLA-DQA1*05:01/DQB1*02:01,HLA-DQA1*05:01/DQB1*03:01,HLA-DQA1*05:01/DQB1*03:01,HLA-DPA1*03:01/DPB1*04:02,HLA-DPA1*03:01/DPB1*04:02,HLA-DQA1*01:02/DQB1*06:02,HLA-DQA1*01:02/DQB1*06:02,HLA-DRB1*04:05,HLA-DRB1*04:05,HLA-DRB3*01:01,HLA-DRB3*01:01,HLA-DRB1*01:01,HLA-DRB1*01:01,HLA-DRB1*09:01,HLA-DRB1*09:01,HLA-DRB1*15:01,HLA-DRB1*15:01 | GTHSWEYWGAQLNAM | Ag85A | 260 | 274 | 0.6034 | - | + | - | - |
| HLA-DQA1*01:01/DQB1*05:01,HLA-DQA1*01:01/DQB1*05:01,HLA-DPA1*01:03/DPB1*04:01,HLA-DPA1*01:03/DPB1*04:01,HLA-DRB1*04:01,HLA-DRB1*04:01,HLA-DPA1*02:01/DPB1*01:01,HLA-DPA1*02:01/DPB1*01:01,HLA-DQA1*05:01/DQB1*03:01,HLA-DQA1*05:01/DQB1*03:01,HLA-DQA1*05:01/DQB1*02:01,HLA-DQA1*05:01/DQB1*02:01,HLA-DPA1*01:03/DPB1*02:01,HLA-DPA1*01:03/DPB1*02:01,HLA-DPA1*03:01/DPB1*04:02,HLA-DPA1*03:01/DPB1*04:02,HLA-DQA1*01:02/DQB1*06:02,HLA-DQA1*01:02/DQB1*06:02,HLA-DRB1*04:05,HLA-DRB1*04:05,HLA-DRB5*01:01,HLA-DRB5*01:01,HLA-DRB3*01:01,HLA-DRB3*01:01,HLA-DRB1*15:01,HLA-DRB1*15:01,HLA-DPA1*02:01/DPB1*05:01,HLA-DPA1*02:01/DPB1*05:01,HLA-DRB1*01:01,HLA-DRB1*01:01,HLA-DRB1*09:01,HLA-DRB1*09:01 | THSWEYWGAQLNAMK | Ag85A | 261 | 275 | 0.5427 | + | + | - | - |
| HLA-DQA1*01:01/DQB1*05:01,HLA-DPA1*01:03/DPB1*04:01,HLA-DRB1*04:01,HLA-DPA1*02:01/DPB1*01:01,HLA-DQA1*05:01/DQB1*03:01,HLA-DQA1*05:01/DQB1*02:01,HLA-DPA1*01:03/DPB1*02:01,HLA-DRB5*01:01,HLA-DPA1*03:01/DPB1*04:02,HLA-DQA1*01:02/DQB1*06:02,HLA-DRB1*04:05,HLA-DRB3*01:01,HLA-DRB1*01:01,HLA-DPA1*02:01/DPB1*05:01,HLA-DRB1*09:01 | HSWEYWGAQLNAMKP | Ag85A | 262 | 276 | 0.5009 | + | + | - | - |
| HLA-DPA1*01:03/DPB1*04:01,HLA-DRB1*04:01,HLA-DPA1*02:01/DPB1*01:01,HLA-DQA1*01:01/DQB1*05:01,HLA-DQA1*05:01/DQB1*03:01,HLA-DRB5*01:01,HLA-DQA1*05:01/DQB1*02:01,HLA-DPA1*01:03/DPB1*02:01,HLA-DQA1*01:02/DQB1*06:02,HLA-DPA1*03:01/DPB1*04:02,HLA-DRB1*04:05,HLA-DRB3*01:01,HLA-DRB1*01:01,HLA-DPA1*02:01/DPB1*05:01,HLA-DRB1*09:01 | SWEYWGAQLNAMKPD | Ag85A | 263 | 277 | 0.8406 | + | + | - | - |
| HLA-DRB1*04:01,HLA-DRB5*01:01,HLA-DQA1*05:01/DQB1*03:01,HLA-DQA1*01:02/DQB1*06:02,HLA-DQA1*05:01/DQB1*02:01,HLA-DPA1*03:01/DPB1*04:02,HLA-DRB1*04:05,HLA-DRB1*09:01,HLA-DPA1*02:01/DPB1*01:01,HLA-DRB1*01:01 | WEYWGAQLNAMKPDL | Ag85A | 264 | 278 | 1.0081 | + | + | - | - |
| HLA-DRB4*01:01,HLA-DRB1*04:01,HLA-DRB5*01:01,HLA-DRB1*04:05,HLA-DQA1*01:02/DQB1*06:02,HLA-DRB1*09:01,HLA-DQA1*05:01/DQB1*03:01,HLA-DQA1*05:01/DQB1*02:01,HLA-DRB1*01:01,HLA-DPA1*03:01/DPB1*04:02 | EYWGAQLNAMKPDLQ | Ag85A | 265 | 279 | 1.0358 | + | + | - | - |
| HLA-DRB4*01:01,HLA-DRB1*04:01,HLA-DRB5*01:01,HLA-DRB1*04:05,HLA-DRB1*01:01,HLA-DQA1*01:02/DQB1*06:02,HLA-DRB1*09:01,HLA-DQA1*05:01/DQB1*03:01 | YWGAQLNAMKPDLQR | Ag85A | 266 | 280 | 0.8196 | + | + | - | - |
| HLA-DRB3*01:01,HLA-DRB4*01:01,HLA-DRB1*01:01 | LNAMKPDLQRALGAT | Ag85A | 271 | 285 | 0.4623 | + | - | - | - |
| HLA-DQA1*05:01/DQB1*03:01,HLA-DRB1*04:01,HLA-DRB1*01:01,HLA-DRB1*09:01,HLA-DRB1*04:05 | KPDLQRALGATPNTG | Ag85A | 275 | 289 | 0.6531 | + | - | - | - |
| HLA-DQA1*05:01/DQB1*03:01,HLA-DRB1*01:01 | LQRALGATPNTGPAP | Ag85A | 278 | 292 | 0.4135 | - | + | - | - |
| HLA-DQA1*05:01/DQB1*03:01 | QRALGATPNTGPAPQ | Ag85A | 279 | 293 | 0.6725 | + | + | - | - |
| HLA-DQA1*05:01/DQB1*03:01,HLA-DQA1*01:02/DQB1*06:02,HLA-DRB1*01:01,HLA-DPA1*03:01/DPB1*04:02,HLA-DRB1*09:01 | GTAAAVVLPGLVGLA | Ag85B | 309 | 323 | 0.4388 | + | - | - | - |
| HLA-DRB1*01:01,HLA-DQA1*05:01/DQB1*03:01,HLA-DRB1*15:01,HLA-DRB1*09:01 | VVLPGLVGLAGGAAT | Ag85B | 314 | 328 | 0.491 | + | - | - | - |
| HLA-DQA1*05:01/DQB1*03:01,HLA-DRB1*01:01,HLA-DRB1*15:01,HLA-DRB1*09:01 | VLPGLVGLAGGAATA | Ag85B | 315 | 329 | 0.5009 | + | - | - | - |
| HLA-DQA1*05:01/DQB1*03:01,HLA-DRB1*01:01,HLA-DRB1*15:01,HLA-DRB1*09:01 | LPGLVGLAGGAATAG | Ag85B | 316 | 330 | 0.7711 | + | - | - | - |
| HLA-DQA1*05:01/DQB1*03:01,HLA-DRB1*01:01,HLA-DRB1*15:01,HLA-DQA1*01:02/DQB1*06:02,HLA-DRB1*09:01 | PGLVGLAGGAATAGA | Ag85B | 317 | 331 | 0.8938 | + | - | - | - |
| HLA-DQA1*05:01/DQB1*03:01,HLA-DRB1*01:01,HLA-DQA1*01:02/DQB1*06:02,HLA-DRB1*09:01 | GLVGLAGGAATAGAF | Ag85B | 318 | 332 | 0.4354 | + | - | - | - |
| HLA-DQA1*05:01/DQB1*03:01,HLA-DRB1*01:01,HLA-DQA1*01:02/DQB1*06:02 | LVGLAGGAATAGAFS | Ag85B | 319 | 333 | 0.5711 | + | - | - | - |
| HLA-DQA1*05:01/DQB1*03:01,HLA-DQA1*01:02/DQB1*06:02,HLA-DRB1*01:01 | VGLAGGAATAGAFSR | Ag85B | 320 | 334 | 0.6323 | + | - | - | - |
| HLA-DQA1*05:01/DQB1*03:01,HLA-DQA1*01:02/DQB1*06:02,HLA-DRB1*01:01 | GLAGGAATAGAFSRP | Ag85B | 321 | 335 | 0.5651 | + | - | - | - |
| HLA-DQA1*05:01/DQB1*03:01,HLA-DQA1*01:02/DQB1*06:02,HLA-DRB1*01:01 | LAGGAATAGAFSRPG | Ag85B | 322 | 336 | 0.5091 | + | - | - | - |
| HLA-DQA1*05:01/DQB1*03:01,HLA-DQA1*01:02/DQB1*06:02 | AGGAATAGAFSRPGL | Ag85B | 323 | 337 | 0.6175 | + | - | - | - |
| HLA-DQA1*01:02/DQB1*06:02,HLA-DQA1*05:01/DQB1*03:01,HLA-DRB1*01:01 | SGGNNSPAVYLLDGL | Ag85B | 359 | 373 | 0.6609 | + | - | - | - |
| HLA-DRB3*01:01,HLA-DRB1*04:05,HLA-DRB1*01:01,HLA-DRB1*12:01,HLA-DRB4*01:01 | VYLLDGLRAQDDYNG | Ag85B | 367 | 381 | 0.5788 | + | - | - | - |
| HLA-DQA1*01:01/DQB1*05:01 | LRAQDDYNGWDINTP | Ag85B | 373 | 387 | 1.2208 | + | - | - | - |
| HLA-DPA1*01:03/DPB1*02:01 | GWDINTPAFEWYYQS | Ag85B | 381 | 395 | 1.7344 | + | + | - | - |
| HLA-DPA1*01:03/DPB1*02:01,HLA-DPA1*02:01/DPB1*01:01,HLA-DRB1*15:01,HLA-DRB1*09:01,HLA-DPA1*03:01/DPB1*04:02 | INTPAFEWYYQSGLS | Ag85B | 384 | 398 | 1.1806 | + | + | - | - |
| HLA-DPA1*01:03/DPB1*02:01,HLA-DQA1*01:01/DQB1*05:01,HLA-DRB1*09:01,HLA-DPA1*01:03/DPB1*04:01,HLA-DRB1*01:01,HLA-DPA1*02:01/DPB1*01:01,HLA-DPA1*03:01/DPB1*04:02,HLA-DQA1*05:01/DQB1*02:01,HLA-DRB1*15:01,HLA-DRB1*04:05,HLA-DRB1*07:01,HLA-DRB3*01:01 | NTPAFEWYYQSGLSI | Ag85B | 385 | 399 | 1.1043 | + | + | - | - |
| HLA-DPA1*01:03/DPB1*04:01,HLA-DPA1*03:01/DPB1*04:02,HLA-DPA1*01:03/DPB1*02:01,HLA-DRB1*09:01,HLA-DPA1*02:01/DPB1*01:01,HLA-DQA1*01:01/DQB1*05:01,HLA-DQA1*05:01/DQB1*02:01,HLA-DRB1*07:01,HLA-DRB1*01:01,HLA-DRB1*15:01,HLA-DRB1*04:05,HLA-DRB1*13:02,HLA-DRB1*04:01,HLA-DRB5*01:01,HLA-DRB3*01:01 | TPAFEWYYQSGLSIV | Ag85B | 386 | 400 | 0.9218 | + | + | - | - |
| HLA-DRB1*09:01,HLA-DPA1*01:03/DPB1*04:01,HLA-DPA1*03:01/DPB1*04:02,HLA-DRB1*04:01,HLA-DPA1*01:03/DPB1*02:01,HLA-DPA1*02:01/DPB1*01:01,HLA-DRB1*07:01,HLA-DRB1*01:01,HLA-DRB3*02:02,HLA-DQA1*01:01/DQB1*05:01,HLA-DQA1*05:01/DQB1*02:01,HLA-DRB1*04:05,HLA-DRB1*15:01,HLA-DRB5*01:01,HLA-DQA1*05:01/DQB1*03:01,HLA-DRB1*13:02,HLA-DRB3*01:01 | PAFEWYYQSGLSIVM | Ag85B | 387 | 401 | 1.0212 | + | + | - | - |
| HLA-DQA1*01:02/DQB1*06:02,HLA-DRB4*01:01,HLA-DQA1*05:01/DQB1*03:01,HLA-DRB1*11:01,HLA-DRB1*01:01,HLA-DRB1*09:01 | QSGLSIVMPVGGQSS | Ag85B | 394 | 408 | 0.8785 | + | - | - | + |
| HLA-DRB4*01:01,HLA-DRB1*15:01,HLA-DQA1*05:01/DQB1*03:01,HLA-DRB1*01:01,HLA-DRB1*11:01,HLA-DRB1*09:01 | SGLSIVMPVGGQSSF | Ag85B | 395 | 409 | 0.5031 | - | - | - | + |
| HLA-DQA1*01:01/DQB1*05:01,HLA-DPA1*01:03/DPB1*02:01,HLA-DRB3*01:01,HLA-DRB1*04:01 | SSFYSDWYSPACGKA | Ag85B | 407 | 421 | 0.5411 | + | + | - | - |
| HLA-DQA1*01:01/DQB1*05:01,HLA-DPA1*01:03/DPB1*02:01,HLA-DQA1*05:01/DQB1*03:01,HLA-DRB1*04:01 | SFYSDWYSPACGKAG | Ag85B | 408 | 422 | 0.9294 | + | + | - | - |
| HLA-DRB1*07:01,HLA-DRB1*13:02,HLA-DQA1*05:01/DQB1*03:01,HLA-DRB1*09:01,HLA-DRB1*01:01 | LSANRAVKPTGSAAI | Ag85B | 440 | 454 | 0.4218 | + | + | - | - |
| HLA-DRB1*07:01,HLA-DRB1*09:01,HLA-DQA1*05:01/DQB1*03:01,HLA-DRB1*08:02,HLA-DRB1*13:02,HLA-DRB1*04:01,HLA-DRB1*01:01 | SANRAVKPTGSAAIG | Ag85B | 441 | 455 | 0.5767 | + | - | - | - |
| HLA-DRB1*07:01,HLA-DRB1*09:01,HLA-DRB1*13:02,HLA-DQA1*05:01/DQB1*03:01,HLA-DRB1*08:02,HLA-DRB1*04:01,HLA-DRB1*01:01,HLA-DRB1*15:01 | ANRAVKPTGSAAIGL | Ag85B | 442 | 456 | 0.554 | + | - | - | - |
| HLA-DQA1*05:01/DQB1*03:01,HLA-DRB1*07:01,HLA-DRB1*09:01,HLA-DRB1*13:02,HLA-DRB1*08:02,HLA-DRB1*04:01,HLA-DRB1*01:01,HLA-DRB1*15:01 | NRAVKPTGSAAIGLS | Ag85B | 443 | 457 | 0.607 | + | - | - | - |
| HLA-DQA1*05:01/DQB1*03:01,HLA-DRB1*07:01,HLA-DRB1*09:01,HLA-DRB1*13:02,HLA-DQA1*01:02/DQB1*06:02,HLA-DRB1*08:02,HLA-DRB1*04:01,HLA-DRB1*15:01,HLA-DRB1*01:01 | RAVKPTGSAAIGLSM | Ag85B | 444 | 458 | 0.744 | + | - | - | - |
| HLA-DQA1*05:01/DQB1*03:01,HLA-DQA1*01:02/DQB1*06:02,HLA-DRB1*07:01,HLA-DRB1*09:01,HLA-DRB1*13:02,HLA-DRB1*01:01 | AVKPTGSAAIGLSMA | Ag85B | 445 | 459 | 0.794 | + | - | - | - |
| HLA-DQA1*05:01/DQB1*03:01,HLA-DQA1*01:02/DQB1*06:02,HLA-DRB1*01:01,HLA-DRB1*09:01 | KPTGSAAIGLSMAGS | Ag85B | 447 | 461 | 0.9162 | + | - | - | - |
| HLA-DQA1*05:01/DQB1*03:01,HLA-DQA1*01:02/DQB1*06:02,HLA-DRB1*09:01,HLA-DRB1*01:01 | PTGSAAIGLSMAGSS | Ag85B | 448 | 462 | 1.0429 | + | - | - | - |
| HLA-DQA1*05:01/DQB1*03:01,HLA-DQA1*01:02/DQB1*06:02,HLA-DRB1*01:01,HLA-DRB1*09:01 | TGSAAIGLSMAGSSA | Ag85B | 449 | 463 | 1.2409 | + | - | - | - |
| HLA-DQA1*05:01/DQB1*03:01,HLA-DRB1*09:01,HLA-DQA1*01:02/DQB1*06:02,HLA-DRB1*01:01,HLA-DRB1*07:01,HLA-DRB1*04:01 | GSAAIGLSMAGSSAM | Ag85B | 450 | 464 | 0.9638 | + | - | - | - |
| HLA-DQA1*05:01/DQB1*03:01,HLA-DRB1*09:01,HLA-DQA1*01:02/DQB1*06:02,HLA-DRB1*01:01,HLA-DRB1*07:01,HLA-DRB1*04:01,HLA-DRB5*01:01 | SAAIGLSMAGSSAMI | Ag85B | 451 | 465 | 0.863 | + | - | - | - |
| HLA-DQA1*05:01/DQB1*03:01,HLA-DRB1*09:01,HLA-DRB1*07:01,HLA-DQA1*01:02/DQB1*06:02,HLA-DRB3*02:02,HLA-DRB1*01:01,HLA-DRB1*04:01,HLA-DRB1*13:02,HLA-DRB1*15:01,HLA-DRB5*01:01,HLA-DPA1*03:01/DPB1*04:02 | AAIGLSMAGSSAMIL | Ag85B | 452 | 466 | 0.7671 | + | - | - | - |
| HLA-DQA1*05:01/DQB1*03:01,HLA-DQA1*01:02/DQB1*06:02,HLA-DRB1*09:01,HLA-DRB1*07:01,HLA-DRB3*02:02,HLA-DRB1*01:01,HLA-DRB1*04:01,HLA-DRB1*13:02,HLA-DRB1*15:01,HLA-DPA1*03:01/DPB1*04:02,HLA-DRB5*01:01 | AIGLSMAGSSAMILA | Ag85B | 453 | 467 | 0.7778 | + | - | - | - |
| HLA-DQA1*01:02/DQB1*06:02,HLA-DRB1*09:01,HLA-DQA1*05:01/DQB1*03:01,HLA-DRB1*07:01,HLA-DRB1*01:01,HLA-DRB1*13:02,HLA-DPA1*03:01/DPB1*04:02,HLA-DRB1*15:01,HLA-DRB1*12:01 | GLSMAGSSAMILAAY | Ag85B | 455 | 469 | 0.7257 | + | - | - | - |
| HLA-DQA1*01:02/DQB1*06:02,HLA-DQA1*05:01/DQB1*03:01,HLA-DRB1*09:01,HLA-DRB1*07:01,HLA-DRB1*01:01,HLA-DPA1*03:01/DPB1*04:02,HLA-DRB5*01:01,HLA-DRB1*12:01 | LSMAGSSAMILAAYH | Ag85B | 456 | 470 | 0.5612 | + | - | - | - |
| HLA-DQA1*01:02/DQB1*06:02,HLA-DQA1*05:01/DQB1*03:01,HLA-DRB1*15:01,HLA-DRB1*12:01,HLA-DRB1*07:01,HLA-DRB1*01:01,HLA-DRB1*09:01,HLA-DRB5*01:01 | SMAGSSAMILAAYHP | Ag85B | 457 | 471 | 0.8015 | + | - | - | - |
| HLA-DQA1*01:02/DQB1*06:02,HLA-DQA1*05:01/DQB1*03:01,HLA-DRB1*15:01,HLA-DRB1*12:01,HLA-DRB1*09:01,HLA-DRB5*01:01,HLA-DRB1*01:01,HLA-DRB1*07:01 | MAGSSAMILAAYHPQ | Ag85B | 458 | 472 | 0.7655 | + | - | - | + |
| HLA-DQA1*01:02/DQB1*06:02,HLA-DQA1*05:01/DQB1*03:01,HLA-DRB1*15:01,HLA-DRB1*12:01,HLA-DRB4*01:01,HLA-DRB1*09:01,HLA-DRB5*01:01,HLA-DRB1*08:02,HLA-DRB1*11:01,HLA-DRB1*01:01 | AGSSAMILAAYHPQQ | Ag85B | 459 | 473 | 0.7543 | - | - | - | + |
| HLA-DQA1*01:02/DQB1*06:02,HLA-DQA1*05:01/DQB1*03:01,HLA-DRB4*01:01,HLA-DRB1*15:01,HLA-DRB1*09:01,HLA-DRB1*12:01,HLA-DRB1*08:02,HLA-DRB5*01:01,HLA-DRB1*07:01,HLA-DRB1*11:01,HLA-DRB1*04:05,HLA-DRB1*01:01,HLA-DRB1*04:01 | GSSAMILAAYHPQQF | Ag85B | 460 | 474 | 0.549 | - | - | - | + |
| HLA-DRB1*01:01 | LSALLDPSQGMGPSL | Ag85B | 480 | 494 | 0.5028 | + | - | - | - |
| HLA-DQA1*05:01/DQB1*03:01,HLA-DRB1*01:01 | ALLDPSQGMGPSLIG | Ag85B | 482 | 496 | 0.475 | + | - | - | - |
| HLA-DQA1*05:01/DQB1*03:01,HLA-DRB1*01:01 | LLDPSQGMGPSLIGL | Ag85B | 483 | 497 | 0.5054 | + | - | + | - |
| HLA-DQA1*05:01/DQB1*03:01,HLA-DRB1*09:01,HLA-DRB1*01:01 | LDPSQGMGPSLIGLA | Ag85B | 484 | 498 | 0.7432 | + | - | - | - |
| HLA-DQA1*05:01/DQB1*03:01,HLA-DRB1*09:01,HLA-DRB1*01:01 | DPSQGMGPSLIGLAM | Ag85B | 485 | 499 | 0.9049 | + | - | - | - |
| HLA-DQA1*05:01/DQB1*03:01,HLA-DRB1*09:01,HLA-DRB1*01:01 | PSQGMGPSLIGLAMG | Ag85B | 486 | 500 | 0.7997 | + | - | - | - |
| HLA-DQA1*01:02/DQB1*06:02,HLA-DQA1*05:01/DQB1*03:01,HLA-DRB1*01:01,HLA-DRB1*09:01 | SQGMGPSLIGLAMGD | Ag85B | 487 | 501 | 0.6056 | + | - | - | - |
| HLA-DQA1*05:01/DQB1*03:01 | AMGDAGGYKAADMWG | Ag85B | 498 | 512 | 0.7173 | - | + | - | - |
| HLA-DQA1*05:01/DQB1*03:01,HLA-DQA1*01:02/DQB1*06:02 | GGYKAADMWGPSSDP | Ag85B | 503 | 517 | 0.4563 | - | + | - | - |
| HLA-DQA1*05:01/DQB1*03:01 | GYKAADMWGPSSDPA | Ag85B | 504 | 518 | 0.436 | - | + | - | - |
| HLA-DQA1*05:01/DQB1*03:01,HLA-DQA1*01:02/DQB1*06:02,HLA-DQA1*04:01/DQB1*04:02 | NGTPNELGGANIPAE | Ag85B | 544 | 558 | 1.0454 | - | + | - | - |
| HLA-DQA1*04:01/DQB1*04:02,HLA-DQA1*05:01/DQB1*03:01,HLA-DQA1*01:02/DQB1*06:02,HLA-DQA1*03:01/DQB1*03:02 | GTPNELGGANIPAEF | Ag85B | 545 | 559 | 0.865 | - | + | - | - |
| HLA-DQA1*04:01/DQB1*04:02,HLA-DQA1*03:01/DQB1*03:02,HLA-DQA1*05:01/DQB1*03:01,HLA-DQA1*01:02/DQB1*06:02,HLA-DQA1*05:01/DQB1*02:01,HLA-DRB1*01:01 | TPNELGGANIPAEFL | Ag85B | 546 | 560 | 0.7388 | - | + | - | - |
| HLA-DQA1*04:01/DQB1*04:02,HLA-DQA1*03:01/DQB1*03:02,HLA-DQA1*05:01/DQB1*03:01,HLA-DQA1*01:02/DQB1*06:02,HLA-DQA1*05:01/DQB1*02:01,HLA-DRB1*01:01 | PNELGGANIPAEFLE | Ag85B | 547 | 561 | 0.561 | - | + | - | - |
| HLA-DQA1*04:01/DQB1*04:02,HLA-DQA1*03:01/DQB1*03:02,HLA-DQA1*05:01/DQB1*03:01,HLA-DQA1*05:01/DQB1*02:01,HLA-DQA1*01:02/DQB1*06:02,HLA-DRB1*01:01 | NELGGANIPAEFLEN | Ag85B | 548 | 562 | 0.4357 | - | + | - | - |
| HLA-DRB1*13:02,HLA-DRB3*02:02,HLA-DRB1*04:01,HLA-DRB5*01:01,HLA-DRB3*01:01,HLA-DRB1*09:01,HLA-DRB1*04:05,HLA-DRB1*11:01,HLA-DRB1*15:01,HLA-DPA1*02:01/DPB1*05:01,HLA-DRB1*08:02,HLA-DRB1*03:01,HLA-DRB4*01:01,HLA-DPA1*01:03/DPB1*02:01,HLA-DRB1*07:01,HLA-DRB1*12:01,HLA-DRB1*01:01 | LENFVRSSNLKFQDA | Ag85B | 560 | 574 | 0.6214 | + | + | - | - |
| HLA-DRB1*13:02,HLA-DRB3*02:02,HLA-DRB1*04:01,HLA-DRB3*01:01,HLA-DRB5*01:01,HLA-DRB1*09:01,HLA-DRB1*04:05,HLA-DRB1*11:01,HLA-DRB1*15:01,HLA-DRB1*08:02,HLA-DPA1*02:01/DPB1*05:01,HLA-DRB4*01:01,HLA-DRB1*03:01,HLA-DRB1*07:01,HLA-DPA1*01:03/DPB1*02:01,HLA-DRB1*01:01 | ENFVRSSNLKFQDAY | Ag85B | 561 | 575 | 0.9024 | + | + | - | - |
| HLA-DRB3*02:02,HLA-DRB1*13:02,HLA-DRB1*04:01,HLA-DRB5*01:01,HLA-DRB1*11:01,HLA-DRB1*15:01,HLA-DRB3*01:01,HLA-DRB1*08:02,HLA-DRB1*04:05,HLA-DRB1*03:01,HLA-DRB1*09:01,HLA-DRB4*01:01,HLA-DRB1*07:01,HLA-DRB1*01:01 | NFVRSSNLKFQDAYN | Ag85B | 562 | 576 | 0.9528 | + | + | - | - |
| HLA-DRB1*04:01,HLA-DRB1*13:02,HLA-DRB1*11:01,HLA-DRB1*15:01,HLA-DRB5*01:01,HLA-DRB4*01:01,HLA-DRB1*03:01,HLA-DRB1*09:01,HLA-DRB1*07:01,HLA-DRB1*01:01 | FVRSSNLKFQDAYNA | Ag85B | 563 | 577 | 1.0317 | + | + | - | - |
| HLA-DRB1*15:01,HLA-DRB4*01:01,HLA-DRB1*04:01,HLA-DRB1*08:02,HLA-DRB1*09:01,HLA-DRB1*01:01 | VRSSNLKFQDAYNAA | Ag85B | 564 | 578 | 1.3694 | + | + | - | - |
| HLA-DRB1*04:01,HLA-DQA1*05:01/DQB1*03:01,HLA-DRB5*01:01,HLA-DRB1*01:01,HLA-DRB1*09:01,HLA-DRB1*08:02 | NLKFQDAYNAAGGHN | Ag85B | 568 | 582 | 1.736 | - | + | - | - |
| HLA-DRB1*04:01,HLA-DRB1*09:01,HLA-DRB1*01:01,HLA-DQA1*05:01/DQB1*03:01,HLA-DRB5*01:01 | LKFQDAYNAAGGHNA | Ag85B | 569 | 583 | 1.4968 | - | + | - | - |
| HLA-DQA1*05:01/DQB1*03:01 | AAGGHNAVFNFPPNG | Ag85B | 577 | 591 | 1.1619 | - | - | + | - |
| HLA-DRB1*15:01,HLA-DRB5*01:01,HLA-DRB1*01:01 | GGHNAVFNFPPNGTH | Ag85B | 579 | 593 | 1.0575 | - | - | + | - |
| HLA-DQA1*01:01/DQB1*05:01,HLA-DPA1*01:03/DPB1*02:01,HLA-DRB1*09:01 | PNGTHSWEYWGAQLN | Ag85B | 589 | 603 | 0.5137 | - | + | - | - |
| HLA-DQA1*01:01/DQB1*05:01,HLA-DPA1*01:03/DPB1*02:01,HLA-DRB1*09:01 | NGTHSWEYWGAQLNA | Ag85B | 590 | 604 | 0.6702 | - | + | - | - |
| HLA-DQA1*01:01/DQB1*05:01,HLA-DPA1*01:03/DPB1*04:01,HLA-DRB1*04:01,HLA-DPA1*02:01/DPB1*01:01,HLA-DQA1*05:01/DQB1*03:01,HLA-DRB5*01:01,HLA-DPA1*01:03/DPB1*02:01,HLA-DPA1*03:01/DPB1*04:02,HLA-DQA1*01:02/DQB1*06:02,HLA-DRB1*04:05,HLA-DRB1*01:01,HLA-DRB3*01:01,HLA-DPA1*02:01/DPB1*05:01,HLA-DRB1*09:01 | HSWEYWGAQLNAMKG | Ag85B | 593 | 607 | 0.4379 | + | + | - | - |
| HLA-DRB1*04:01,HLA-DPA1*02:01/DPB1*01:01,HLA-DRB5*01:01,HLA-DQA1*05:01/DQB1*03:01,HLA-DQA1*01:01/DQB1*05:01,HLA-DPA1*01:03/DPB1*02:01,HLA-DQA1*01:02/DQB1*06:02,HLA-DPA1*03:01/DPB1*04:02,HLA-DRB1*04:05,HLA-DRB1*01:01,HLA-DRB3*01:01,HLA-DPA1*02:01/DPB1*05:01,HLA-DRB1*09:01 | SWEYWGAQLNAMKGD | Ag85B | 594 | 608 | 0.8242 | + | + | - | - |
| HLA-DRB1*04:01,HLA-DRB5*01:01,HLA-DQA1*05:01/DQB1*03:01,HLA-DQA1*01:02/DQB1*06:02,HLA-DPA1*03:01/DPB1*04:02,HLA-DRB1*01:01,HLA-DPA1*02:01/DPB1*01:01,HLA-DRB1*09:01 | WEYWGAQLNAMKGDL | Ag85B | 595 | 609 | 0.7147 | + | + | - | - |
| HLA-DRB1*04:01,HLA-DRB5*01:01,HLA-DQA1*01:02/DQB1*06:02,HLA-DRB1*04:05,HLA-DQA1*05:01/DQB1*03:01,HLA-DRB1*01:01,HLA-DRB4*01:01,HLA-DRB1*09:01,HLA-DPA1*03:01/DPB1*04:02 | EYWGAQLNAMKGDLQ | Ag85B | 596 | 610 | 0.7542 | + | + | - | - |
| HLA-DQA1*03:01/DQB1*03:02,HLA-DQA1*04:01/DQB1*04:02,HLA-DQA1*05:01/DQB1*02:01,HLA-DRB1*01:01 | ARRPQNLLDVTAEPA | Rv1733c | 642 | 656 | 0.5401 | - | - | - | + |
| HLA-DRB5*01:01,HLA-DQA1*05:01/DQB1*03:01,HLA-DRB1*01:01 | LLDVTAEPARGRKRT | Rv1733c | 648 | 662 | 0.9585 | - | - | - | + |
| HLA-DRB5*01:01,HLA-DQA1*05:01/DQB1*03:01 | LDVTAEPARGRKRTL | Rv1733c | 649 | 663 | 1.0448 | - | + | - | + |
| HLA-DRB5*01:01,HLA-DQA1*05:01/DQB1*03:01 | DVTAEPARGRKRTLS | Rv1733c | 650 | 664 | 1.1329 | - | + | - | + |
| HLA-DRB5*01:01 | VTAEPARGRKRTLSD | Rv1733c | 651 | 665 | 0.9786 | - | + | - | + |
| HLA-DQA1*05:01/DQB1*03:01,HLA-DRB1*03:01,HLA-DRB1*13:02,HLA-DRB1*07:01 | AAGTAVQDSRSHVYA | Rv1733c | 682 | 696 | 0.6868 | + | - | - | + |
| HLA-DRB1*03:01,HLA-DRB1*13:02,HLA-DRB1*07:01 | AGTAVQDSRSHVYAH | Rv1733c | 683 | 697 | 0.5733 | + | - | - | + |
