# Supplementary material for: Bioinformatics Analysis and Immunogenicity Assessment of the Novel Multi‐Stage DNA Vaccine W541 Against Mycobacterium Tuberculosis
Source: Immun Inflamm Dis. 2024 Nov 26;12(11):e70074. doi: 10.1002/iid3.70074 (PMC11590035; doi:10.1002/iid3.70074)
Supplement: Supplementary file 3 — Supporting information. [file IID3-12-e70074-s003.docx]

**Supplementary Table 3 Predicted CTL epitopes of W541 vaccine protein**

| allele | peptide | source | start | end | length | antigenicity | Class I Immunogenicity |
| --- | --- | --- | --- | --- | --- | --- | --- |
| HLA-B*07:02,HLA-B*07:02 | RPGLPVEYL | Ag85A | 3 | 11 | 9 | 0.5177 | 0.11024 |
| HLA-A*02:03,HLA-A*02:03,HLA-A*02:01,HLA-A*02:01 | GLPVEYLQV | Ag85A | 5 | 13 | 9 | 0.7069 | 0.05492 |
| HLA-B*15:01,HLA-B*15:01 | GLPVEYLQVP | Ag85A | 5 | 14 | 10 | 0.4997 | 0.04776 |
| HLA-B*35:01,HLA-B*35:01 | LPVEYLQVP | Ag85A | 6 | 14 | 9 | 0.7445 | 0.02647 |
| HLA-B*35:01,HLA-B*35:01 | LPVEYLQVPS | Ag85A | 6 | 15 | 10 | 0.6477 | 0.01907 |
| HLA-A*02:06,HLA-A*02:01,HLA-A*68:02,HLA-A*02:03 | FSGWDINTPA | Ag85A | 48 | 57 | 10 | 0.9887 | 0.40528 |
| HLA-A*02:06 | SGWDINTPA | Ag85A | 49 | 57 | 9 | 1.4382 | 0.24401 |
| HLA-A*23:01,HLA-B*15:01 | SGWDINTPAF | Ag85A | 49 | 58 | 10 | 1.5629 | 0.26756 |
| HLA-A*68:02 | NTPAFEWYD | Ag85A | 54 | 62 | 9 | 0.927 | 0.4288 |
| HLA-B*07:02 | KPTGSAVVGL | Ag85A | 116 | 125 | 10 | 0.5141 | 0.0172 |
| HLA-B*58:01,HLA-B*15:01 | GSAVVGLSM | Ag85A | 119 | 127 | 9 | 1.0016 | 0.02032 |
| HLA-A*32:01,HLA-B*58:01 | ASSALTLAI | Ag85A | 129 | 137 | 9 | 0.9517 | 0.02491 |
| HLA-A*30:02 | SSALTLAIYH | Ag85A | 130 | 139 | 10 | 0.6675 | 0.17553 |
| HLA-A*02:03 | ALTLAIYHP | Ag85A | 132 | 140 | 9 | 1.0436 | 0.1806 |
| HLA-A*02:03,HLA-A*02:01,HLA-A*02:06 | AMGPTLIGL | Ag85A | 158 | 166 | 9 | 0.4798 | 0.15932 |
| HLA-A*02:03,HLA-A*02:06 | AMGPTLIGLA | Ag85A | 158 | 167 | 10 | 0.4035 | 0.17424 |
| HLA-B*35:01,HLA-B*07:02,HLA-B*53:01,HLA-B*15:01 | MGPTLIGLAM | Ag85A | 159 | 168 | 10 | 0.524 | 0.19966 |
| HLA-B*07:02,HLA-B*35:01 | GPTLIGLAM | Ag85A | 160 | 168 | 9 | 0.4017 | 0.17644 |
| HLA-A*30:02,HLA-A*30:02,HLA-B*35:01,HLA-B*35:01,HLA-B*15:01,HLA-B*15:01 | LAMGDAGGY | Ag85A | 166 | 174 | 9 | 0.5273 | 0.08393 |
| HLA-B*44:02 | SDMWGPKEDP | Ag85A | 177 | 186 | 10 | 0.4753 | 0.08255 |
| HLA-A*68:02 | TRVWVYCGNG | Ag85A | 205 | 214 | 10 | 0.5057 | 0.24696 |
| HLA-A*03:01,HLA-A*11:01,HLA-A*31:01 | RVWVYCGNGK | Ag85A | 206 | 215 | 10 | 0.7151 | 0.10358 |
| HLA-B*07:02 | KPSDLGGNNL | Ag85A | 215 | 224 | 10 | 1.4062 | 0.01348 |
| HLA-A*03:01 | DLGGNNLPAK | Ag85A | 218 | 227 | 10 | 1.4482 | 0.03556 |
| HLA-B*15:01 | NLPAKFLEGF | Ag85A | 223 | 232 | 10 | 0.6164 | 0.03363 |
| HLA-A*02:06,HLA-A*02:03,HLA-A*68:02 | FQDAYNAGGG | Ag85A | 240 | 249 | 10 | 1.4901 | 0.1219 |
| HLA-A*68:02,HLA-A*02:06,HLA-A*02:03 | YNAGGGHNGV | Ag85A | 244 | 253 | 10 | 2.4651 | 0.15759 |
| HLA-A*68:02 | NAGGGHNGV | Ag85A | 245 | 253 | 9 | 3.0386 | 0.12289 |
| HLA-B*58:01 | DSGTHSWEYW | Ag85A | 258 | 267 | 10 | 0.6383 | 0.21131 |
| HLA-B*58:01,HLA-B*57:01 | SGTHSWEYW | Ag85A | 259 | 267 | 9 | 0.4439 | 0.1749 |
| HLA-B*40:01,HLA-B*40:01 | WEYWGAQLN | Ag85A | 264 | 272 | 9 | 0.904 | 0.18728 |
| HLA-A*33:01,HLA-A*33:01 | EYWGAQLNAM | Ag85A | 265 | 274 | 10 | 0.7063 | 0.03826 |
| HLA-A*02:06 | RALGATPNTG | Ag85A | 280 | 289 | 10 | 0.8506 | 0.11318 |
| HLA-A*68:02,HLA-A*02:03,HLA-A*02:06,HLA-A*02:01 | MIGTAAAVV | Ag85B | 307 | 315 | 9 | 0.4617 | 0.18213 |
| HLA-A*68:02 | GTAAAVVLP | Ag85B | 309 | 317 | 9 | 0.9815 | 0.15739 |
| HLA-A*68:02,HLA-A*02:03,HLA-A*02:06 | GTAAAVVLPG | Ag85B | 309 | 318 | 10 | 0.5975 | 0.15339 |
| HLA-A*68:02,HLA-A*02:06,HLA-A*02:03 | TAAAVVLPGL | Ag85B | 310 | 319 | 10 | 0.4792 | 0.13247 |
| HLA-A*02:03,HLA-A*02:06 | AAAVVLPGLV | Ag85B | 311 | 320 | 10 | 0.4378 | 0.09532 |
| HLA-B*15:01 | LVGLAGGAAT | Ag85B | 319 | 328 | 10 | 0.6379 | 0.15872 |
| HLA-A*02:03 | VGLAGGAATA | Ag85B | 320 | 329 | 10 | 1.0136 | 0.1943 |
| HLA-A*02:03,HLA-A*02:01,HLA-A*02:06 | GLAGGAATA | Ag85B | 321 | 329 | 9 | 1.3338 | 0.17233 |
| HLA-B*15:01 | AGGAATAGAF | Ag85B | 323 | 332 | 10 | 1.1862 | 0.21456 |
| HLA-B*15:01 | GGAATAGAF | Ag85B | 324 | 332 | 9 | 1.0961 | 0.17816 |
| HLA-A*68:01 | GAATAGAFSR | Ag85B | 325 | 334 | 10 | 0.4744 | 0.16183 |
| HLA-A*02:06 | AQDDYNGWDI | Ag85B | 375 | 384 | 10 | 0.7214 | 0.25142 |
| HLA-A*68:02 | YSDWYSPACG | Ag85B | 410 | 419 | 10 | 0.4796 | 0.05647 |
| HLA-B*07:02 | KPTGSAAIGL | Ag85B | 447 | 456 | 10 | 0.8471 | 0.09265 |
| HLA-B*58:01,HLA-B*15:01 | TGSAAIGLSM | Ag85B | 449 | 458 | 10 | 1.3825 | 0.07925 |
| HLA-B*58:01,HLA-B*15:01 | GSAAIGLSM | Ag85B | 450 | 458 | 9 | 1.2027 | 0.10755 |
| HLA-A*02:06 | MILAAYHPQ | Ag85B | 464 | 472 | 9 | 0.8702 | 0.09121 |
| HLA-B*07:02,HLA-B*35:01 | GPSLIGLAM | Ag85B | 491 | 499 | 9 | 0.6079 | 0.11014 |
| HLA-A*68:02 | TRLWVYCGNG | Ag85B | 536 | 545 | 10 | 0.5655 | 0.22996 |
| HLA-A*02:03 | RLWVYCGNGT | Ag85B | 537 | 546 | 10 | 0.8136 | 0.10358 |
| HLA-B*35:01 | TPNELGGAN | Ag85B | 546 | 554 | 9 | 1.3143 | 0.17121 |
| HLA-B*07:02 | TPNELGGANI | Ag85B | 546 | 555 | 10 | 1.2042 | 0.18199 |
| HLA-A*68:02 | ELGGANIPA | Ag85B | 549 | 557 | 9 | 0.8819 | 0.18295 |
| HLA-B*15:01 | LGGANIPAEF | Ag85B | 550 | 559 | 10 | 0.4811 | 0.25475 |
| HLA-A*24:02 | AYNAAGGHNA | Ag85B | 574 | 583 | 10 | 1.5728 | 0.16379 |
| HLA-A*68:02,HLA-A*02:03,HLA-A*02:06 | YNAAGGHNAV | Ag85B | 575 | 584 | 10 | 1.6111 | 0.16592 |
| HLA-A*68:02,HLA-B*35:01,HLA-A*02:06,HLA-A*02:03 | NAAGGHNAV | Ag85B | 576 | 584 | 9 | 1.9957 | 0.12765 |
| HLA-B*15:01,HLA-B*35:01 | NAAGGHNAVF | Ag85B | 576 | 585 | 10 | 1.4758 | 0.16235 |
| HLA-B*15:01,HLA-B*35:01 | AAGGHNAVF | Ag85B | 577 | 585 | 9 | 1.1841 | 0.12765 |
| HLA-B*58:01,HLA-A*68:02 | NAVFNFPPNG | Ag85B | 582 | 591 | 10 | 0.6028 | 0.21532 |
| HLA-A*30:01 | AVFNFPPNG | Ag85B | 583 | 591 | 9 | 0.9399 | 0.12191 |
| HLA-A*68:02 | SRYLARVEAG | Rv3407 | 621 | 630 | 10 | 0.4062 | 0.22236 |
| HLA-B*07:02,HLA-B*08:01 | IPARRPQNL | Rv1733c | 640 | 648 | 9 | 0.6312 | 0.0032 |
| HLA-B*07:02 | RPQNLLDVTA | Rv1733c | 644 | 653 | 10 | 0.5649 | 0.01269 |
| HLA-B*07:02 | AEPARGRKRT | Rv1733c | 653 | 662 | 10 | 1.1625 | 0.01613 |
| HLA-B*07:02,HLA-B*08:01 | EPARGRKRTL | Rv1733c | 654 | 663 | 10 | 0.8284 | 0.01154 |
| HLA-B*35:01 | IPFAAAAGT | Rv1733c | 677 | 685 | 9 | 0.7535 | 0.20512 |
| HLA-A*02:06,HLA-A*68:02,HLA-A*02:03 | PFAAAAGTAV | Rv1733c | 678 | 687 | 10 | 0.6259 | 0.21579 |
| HLA-A*02:06,HLA-A*68:02,HLA-B*35:01,HLA-A*02:03,HLA-B*07:02,HLA-A*02:01 | FAAAAGTAV | Rv1733c | 679 | 687 | 9 | 0.6628 | 0.17769 |
| HLA-B*15:01 | FAAAAGTAVQ | Rv1733c | 679 | 688 | 10 | 0.8588 | 0.21685 |
