# Supplementary material for: Bioinformatics Analysis and Immunogenicity Assessment of the Novel Multi‐Stage DNA Vaccine W541 Against Mycobacterium Tuberculosis
Source: Immun Inflamm Dis. 2024 Nov 26;12(11):e70074. doi: 10.1002/iid3.70074 (PMC11590035; doi:10.1002/iid3.70074)
Supplement: Supplementary file 4 — Supporting information. [file IID3-12-e70074-s001.docx]

Supplementary Table 4 Predicted HTL toxic epitopes and CTL toxic epitopes of W541 vaccine protein

|  | peptide | HTL/CTL | source | start | end | length | toxicity | antigenicity |
| --- | --- | --- | --- | --- | --- | --- | --- | --- |
| HLA-DQA1*05:01/DQB1*03:01,HLA-DRB1*01:01 | SDWYQPACGKAGCQT | HTL | Ag85A | 80 | 94 | 15 | + | 1.3236 |
| HLA-DQA1*05:01/DQB1*03:01 | WYQPACGKAGCQTYK | HTL | Ag85A | 82 | 96 | 15 | + | 1.6588 |
| HLA-DQA1*05:01/DQB1*03:01 | YQPACGKAGCQTYKW | HTL | Ag85A | 83 | 97 | 15 | + | 1.6314 |
| HLA-DPA1*01:03/DPB1*02:01,HLA-DPA1*01:03/DPB1*02:01 | ACGKAGCQTYKWETF | HTL | Ag85A | 86 | 100 | 15 | + | 1.2434 |
| HLA-DPA1*01:03/DPB1*04:01,HLA-DPA1*01:03/DPB1*04:01,HLA-DPA1*02:01/DPB1*01:01,HLA-DPA1*02:01/DPB1*01:01,HLA-DPA1*01:03/DPB1*02:01,HLA-DPA1*01:03/DPB1*02:01,HLA-DPA1*03:01/DPB1*04:02,HLA-DPA1*03:01/DPB1*04:02 | CGKAGCQTYKWETFL | HTL | Ag85A | 87 | 101 | 15 | + | 1.1504 |
| HLA-DPA1*01:03/DPB1*04:01,HLA-DPA1*01:03/DPB1*04:01,HLA-DPA1*01:03/DPB1*02:01,HLA-DPA1*01:03/DPB1*02:01,HLA-DPA1*02:01/DPB1*01:01,HLA-DPA1*02:01/DPB1*01:01,HLA-DPA1*02:01/DPB1*05:01,HLA-DPA1*02:01/DPB1*05:01,HLA-DPA1*03:01/DPB1*04:02,HLA-DPA1*03:01/DPB1*04:02 | GKAGCQTYKWETFLT | HTL | Ag85A | 88 | 102 | 15 | + | 0.6963 |
| HLA-DQA1*05:01/DQB1*03:01,HLA-DRB1*01:01 | SDWYSPACGKAGCQT | HTL | Ag85B | 411 | 425 | 15 | + | 1.3085 |
| HLA-DQA1*05:01/DQB1*03:01 | DWYSPACGKAGCQTY | HTL | Ag85B | 412 | 426 | 15 | + | 1.3619 |
| HLA-DQA1*05:01/DQB1*03:01 | WYSPACGKAGCQTYK | HTL | Ag85B | 413 | 427 | 15 | + | 1.5897 |
| HLA-DQA1*05:01/DQB1*03:01 | YSPACGKAGCQTYKW | HTL | Ag85B | 414 | 428 | 15 | + | 1.5705 |
| HLA-B*57:01,HLA-B*57:01,HLA-B*58:01,HLA-B*58:01 | GKAGCQTYKW | CTL | Ag85A | 88 | 97 | 10 | + | 1.5552 |
| HLA-B*57:01,HLA-B*57:01,HLA-B*58:01,HLA-B*58:01,HLA-B*53:01,HLA-B*53:01,HLA-A*32:01,HLA-A*32:01 | KAGCQTYKW | CTL | Ag85A | 89 | 97 | 9 | + | 1.4898 |
| HLA-B*15:01,HLA-B*15:01 | GCQTYKWETF | CTL | Ag85A | 91 | 100 | 10 | + | 0.4826 |
| HLA-B*57:01,HLA-B*57:01,HLA-B*58:01,HLA-B*58:01 | GKAGCQTYKW | CTL | Ag85B | 419 | 428 | 10 | + | 1.5552 |
| HLA-B*57:01,HLA-B*57:01,HLA-B*58:01,HLA-B*58:01,HLA-B*53:01,HLA-B*53:01,HLA-A*32:01,HLA-A*32:01 | KAGCQTYKW | CTL | Ag85B | 420 | 428 | 9 | + | 1.4898 |
| HLA-B*15:01,HLA-B*15:01 | GCQTYKWETF | CTL | Ag85B | 422 | 431 | 10 | + | 0.4826 |
